# Mechanistic Understanding of Nickel Catalyzed Urea Oxidation Reaction to Cyanate and Nitrite

**DOI:** 10.1002/EXP.20240378

**Published:** 2026-06-05

**Authors:** Kyu In Shim, Jiseon Kim, Miyeon Kim, Kangwoo Cho, Jeong Woo Han

**Affiliations:** ^1^ Department of Materials Science and Engineering Research Institute of Advanced Materials Seoul National University Seoul Republic of Korea; ^2^ Division of Environmental Science and Engineering Pohang University of Science and Technology (POSTECH) Pohang Republic of Korea

**Keywords:** density functional theory, electrocatalysis, urea oxidation

## Abstract

The urea oxidation reaction (UOR) is a promising alternative to the oxygen evolution reaction (OER) for sustainable hydrogen production due to its lower onset potential. However, the reasons behind this advantage remain unclear, with inconsistencies in the literature regarding the UOR mechanism. Previously, UOR was mostly believed to proceed via a six‐electron pathway producing N_2_ and CO_2_, but this assumption lacked experimental and theoretical validation. Here, the UOR mechanism is thoroughly re‐evaluated by integrating experimental observations and density functional theory calculations on β‐NiOOH catalyst as a model system. Experimentally, significant UOR current densities of 100 and 500 mA cm^−2^ were achieved at potentials of 1.40 and 1.53 V RHE, respectively, outperforming the OER, which required 1.79 V RHE at 500 mA cm^−2^. Theoretical calculations reveal that oxygen vacancies are thermodynamically favored and serve as preferential adsorption sites for urea, with a significantly lower energy barrier (1.49 eV) compared to the OER (3.25 eV). OCN^−^ and NO_2_^−^ were identified as the primary reaction products, which were also confirmed experimentally. This work not only clarifies the UOR pathway and the critical role of oxygen vacancies in enhancing reaction selectivity and efficiency but also resolves longstanding mechanistic ambiguities, providing a foundation for the rational design of advanced electrocatalysts for efficient hydrogen production and environmental remediation.

## Graphical abstract

The urea oxidation reaction (UOR) is a promising alternative to oxygen evolution reaction (OER) for energy efficient hydrogen production, yet its reaction mechanism remains controversial because many proposed pathways have not been directly correlated with experimentally verified products and intermediates. This study re‐evaluates UOR on β‐NiOOH by integrating DFT‐derived reaction pathways with experimental analyses. The results reveal that oxygen vacancies promote urea adsorption and favor a lower‐energy pathway toward OCN^−^ and NO_2_^−^ formation, challenging the conventional N_2_/CO_2_ pathway and providing mechanistic guidance for designing efficient UOR electrocatalysts.

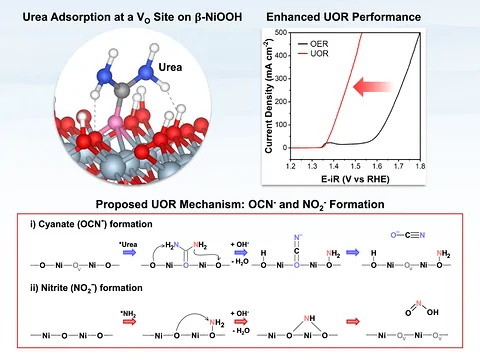

## 1 Introduction

In response to the growing need for sustainable, enduring, and environmentally friendly energy solutions, urea has emerged as a noteworthy player in the realm of nitrogen‐based fuel molecules. Urea, with its chemical formula CO(NH_2_)_2_, is an abundant and versatile compound widely recognized for its pivotal role as a nitrogen‐rich fertilizer in agriculture [1, 2]. This organic molecule, however, brings about significant environmental challenges due to its negative impact on water ecosystems. The runoff of urea‐containing wastewater from agricultural activities contributes to water pollution, fostering eutrophication and posing threats to aquatic ecosystems [3, 4, 5].

Along with the need for urea removal, the urea oxidation reaction (UOR) has been an appealing option due to its application in environmental remediation and energy generation and storage. The UOR, when employed as an alternative to the oxygen evolution reaction (OER), emerges as a potential avenue for sustainable energy solutions. By facilitating the electrochemical conversion of urea, the UOR can drive hydrogen evolution reactions (HER). This process holds promise as a means of harnessing hydrogen as a clean energy source [6, 7, 8, 9]. Typically unfolding in alkaline media, the UOR engages in a six‐electron–proton coupled transfer step, succinctly represented by the equation:

(1) $$\begin{array}{cc} & \mathrm{CO}{\left(NH_{2}\right)}_{2}+6OH^{-}\to N_{2}+CO_{2}+5H_{2}O+6e^{-}\;\\ & \;\left(\;E^{\circ }=0.37\;V\;\mathrm{vs}.\;\mathrm{RHE}\right)\; \end{array}$$

with a standard potential (E°) of 0.37 V (versus the reversible hydrogen electrode (RHE)) [6, 7]. Currently, the UOR grapples with a formidable challenge due to the high overpotential at best ∼1 V, attributed to the intricacies of the 6e^−^ transfer process [7, 8]. Nevertheless, the UOR continues to hold substantial promise as a superior substitute for the OER as the anode half‐reaction in energy‐efficient water electrolyzers for hydrogen production. Additionally, it can serve as the anodic reaction in direct urea fuel cells [10]. Despite the UOR's higher overpotential relative to the OER (the best OER overpotential ∼250 mV for 100 mAcm^−2^), its lower thermodynamic potential (0.37 versus 1.23 V) results in a more favorable overall operational potential [11]. Moreover, the UOR avoids chlorine gas generation, a concern in emerging seawater oxidation (E° = 1.36 V) [12].

Initially, noble metal catalysts such as Pt, Ir, and Ru were utilized as UOR catalysts [7]. However, due to their limited availability and economic impracticality, Ni‐based catalysts were subsequently adopted [13]. Nevertheless, these Ni‐based catalysts exhibit increased overpotentials, reduced current densities, and insufficient stability, highlighting the need for further research and development [14, 15]. For example, one of the most efficient non‐precious UOR catalysts reported to date, a NiCo_2_S_4_@CoMo_2_S_4_ heterojunction grown on carbon cloth, delivers a current density of 10 mA cm^−2^ at a remarkably low potential of 1.15 V in 1.0 M KOH with 0.33 M urea [16]. Regrettably, this improvement remains marginal when juxtaposed with contemporary benchmarks set by OER catalysts onset potential of about 1.45 V [11, 17].

Previously embraced UOR pathway on NiOOH surface involves a series of intermediates, including * → *CO(NH_2_)_2_ → *CO(NH·NH_2_) → *CO(NH·NH) → *CO(NH·N) → *CO(N_2_) → *CO(OH)+N_2_ → *CO(OH·OH) → *COO → CO_2_ [18]. This pathway, computationally developed based on gas‐phase models of NiOOH and urea, was constructed with a specific focus on the production of N_2_ and CO_2_. It attempted to explore alternative reaction pathways, but did not identify other possible products, nor did it comprehensively evaluate the associated energetics. Despite the limitation, this pathway has been widely accepted in the research community, with little scrutiny regarding its assumptions or the exclusion of competing mechanisms. For example, the suggested potential‐determining step (PDS) in this mechanism is the desorption of the *COO intermediate, resulting in a substantial energetic penalty (approximately 1,242.2 kJ mol^−1^) and severely limiting catalytic efficiency due to a strong binding to the Ni^3+^ active sites [7, 8, 12, 18, 19, 20, 21]. Also, the cyanate ion presented the barrier of 6,190 kJ mol^−1^, highlighting that the cyanate ion cannot be separated from NiOOH unless further reactions occur [18]. Most importantly, the above reaction pathway was not validated with experimental results. A lack of critical evaluation of this pathway has contributed to the persistence of gaps in understanding the UOR mechanism and the identification of its true reaction products.

The UOR, characterized by its six‐electron transfer process to produce N_2_ and CO_2_, may offer flexibility beyond the conventional pathway on the NiOOH surface, with reports of alternative mechanisms involving NO*_x_* and CO*_x_* by‐products through multi‐electron transfer reactions [21]. This suggests that the reaction mechanism is not singular but rather encompasses diverse pathways with variable thermodynamics and kinetics. A more nuanced understanding of these mechanisms is critical to unlocking higher catalytic activity, stability, and selectivity. For instance, previous studies propose that novel intermediates with more favorable energetics could significantly improve performance [22, 23].

In this work, we focus on elucidating the mechanistic details of UOR on the β‐NiOOH surface, particularly the roles of oxygen vacancy (V_O_) in modulating reaction pathways. The V_O_ has been shown to alter urea adsorption behaviors and facilitate reaction intermediates by lowering energy barriers [14]. Electrochemical measurements, including linear sweep voltammetry (LSV), Tafel analysis, and electrochemical impedance spectroscopy, were performed to evaluate the kinetics and performance of β‐NiOOH under UOR conditions. Chronopotentiometry (CP) was conducted to assess the stability of the catalyst over time. Structural and compositional changes during the reaction were characterized using Raman spectroscopy and X‐ray photoelectron spectroscopy (XPS), providing critical information on phase transitions to β‐NiOOH, V_O_ formation, and oxidation states of Ni^3+^.

To theoretically elaborate this, we selected a β‐NiOOH phase that maintains the Ni^3+^ charge state through hydrogen distribution and evaluated the influence of surface V_O_ on urea adsorption configurations [24]. Our density functional theory (DFT) calculations along with the reaction pathway demonstrate that β‐NiOOH achieves UOR at significantly lower potentials than OER, as observed in the experimental results. DFT results also identified cyanate (OCN^−^) and nitrite (NO_2_^−^) as the primary reaction products, diverging from the traditionally assumed six‐electron pathway producing N_2_ and CO_2_. These findings were further validated by ion chromatography (IC) and in situ Fourier transform infrared (FT‐IR) spectroscopy, which reveal that urea preferentially adsorbs at V_O_ sites, enabling a novel pathway with more favorable thermodynamics and kinetics to produce OCN^−^ and NO_2_^−^.

## 2 Results and Discussion

A significant factor contributing to the lack of critical evaluation of the UOR pathway is the diverse selection of Ni(OH)_2_ phases employed in studies. Researchers have used various phases, including Ni(OH)_2_, α‐NiOOH, β‐NiOOH, and γ‐NiOOH, each of which can undergo phase transformations under applied voltage due to their electrocatalytic nature [14, 25, 26, 27]. α‐Ni(OH)_2_ is a layered double hydroxide material characterized by the intercalation of water molecules and anions between the Ni(OH)_2_ layers [28]. Through chemical aging of α‐Ni(OH)_2_ in basic electrolyte, β‐Ni(OH)_2_ is formed without interlayer species [29]. Ni with an oxidation state of +2 further undergoes self‐reconstruction into catalytically active β‐NiOOH, where Ni exists in the +3 oxidation state and plays a key role in UOR and OER [27]. Upon the application of anodic potential, β‐NiOOH is transformed into γ‐NiOOH, where water molecules and alkali metal cations are intercalated between the NiO_2_ layers [28, 30]. Additionally, NiOO^−^ species are formed via the deprotonation of NiOOH, and in Fe‐free oxides such as Ni and NiCo oxides, it can exchange with OH^−^ from the electrolyte and is known as an important precursor in the O_2_ desorption step of the OER [31, 32]. These phases differ in their nickel oxidation states, which are crucial for understanding catalytic behavior. Initially, the active phase for UOR was therefore evaluated experimentally. To compare electrochemical activity, a three‐electrode system was used to evaluate Ni(OH)_2_ in 1 M KOH electrolyte with and without 0.33 M urea. Ni(OH)_2_ was synthesized via cathodic deposition (detailed methods are provided in the Experimental section of Supporting Information). LSV curves showed that the potential required for the UOR was lower than that for the OER (Figure 1a). Notably, to achieve significant UOR current densities of 100 mA cm^−2^ and 500 mA cm^−2^, potentials of 1.40 V RHE and 1.53 V RHE were required, respectively, surpassing the OER, which required 1.79 V RHE at 500 mA cm^−2^. Furthermore, Tafel slopes revealed a lower value for UOR at 58.2 mV dec^−1^ compared to 124.1 mV dec^−1^ for OER, clearly indicating inferior kinetics in the OER (Figure 1b). The Nyquist plots were fitted using an equivalent circuit model [33]. The fitted charge transfer resistance (R_ct_) values of 1.386 and 0.241 Ω for OER and UOR, respectively, confirm significantly faster charge transfer kinetics for UOR (Figure 1c). Chronopotentiometry (CP) measurements were conducted at a current density of 100 mA cm^−2^ (Figure S1). The potential remained nearly constant over one hour, demonstrating that the presence of urea in the electrolyte lowers the required energy for the UOR (average potential = 1.42 V; OER = 1.59 V).

**FIGURE 1:**
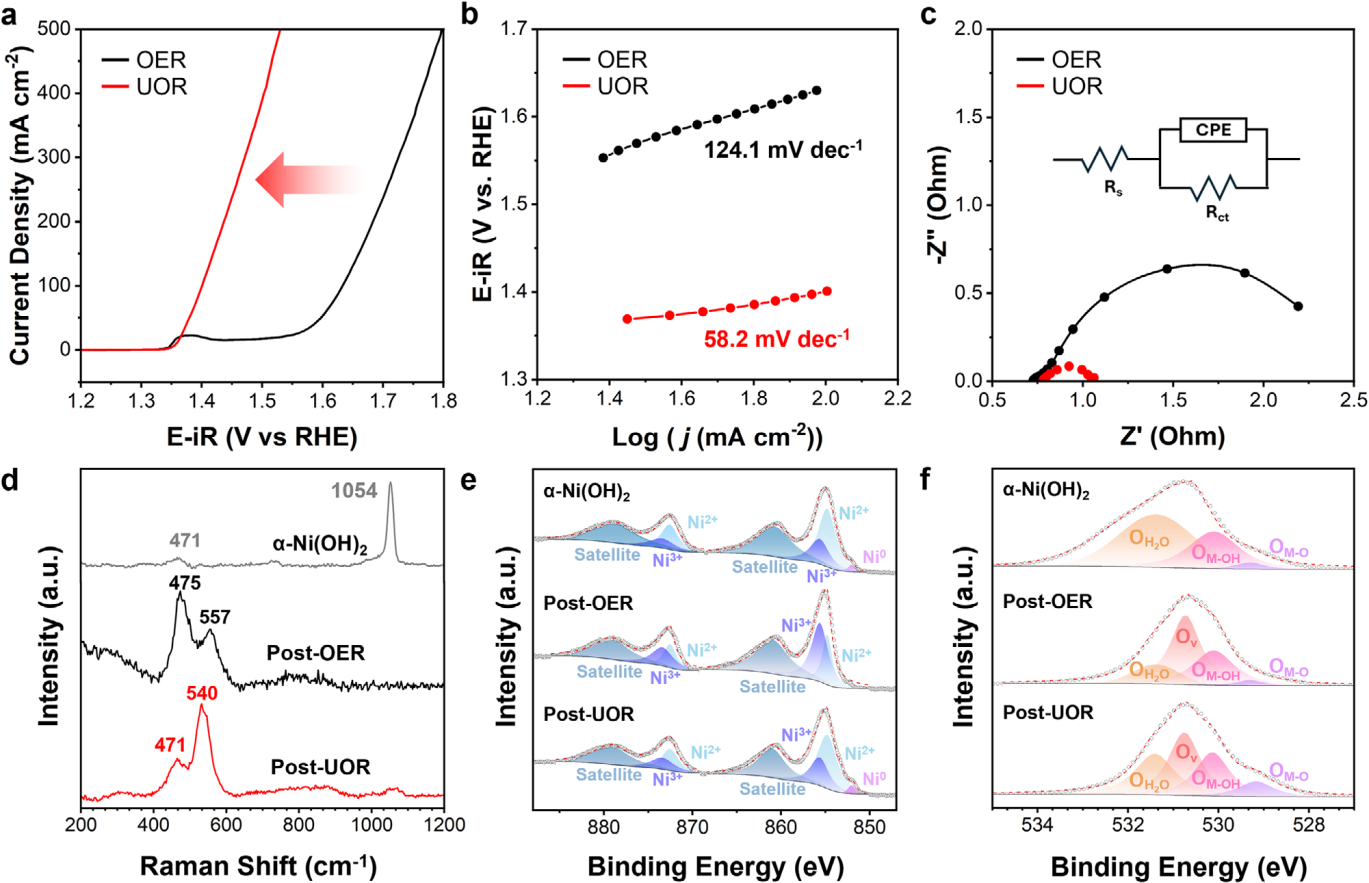
Electrochemical studies of the α‐Ni(OH)_2_ electrode: (a) LSV curves with 85% iR compensation in 1 M KOH and 1 M KOH + 0.33 M urea electrolytes, (b) Tafel plots, and (c) Nyquist plots for OER and UOR. Structural characteristics of the electrode before and after CP measurements: (d) Raman spectra, XPS spectra; (e) Ni 2p; and (f) O 1s.

To confirm the successful synthesis and phase identity of the Ni(OH)_2_ catalyst, we conducted a detailed structural characterization. The X‐ray diffraction (XRD) pattern of the synthesized Ni(OH)_2_ catalyst (Figure **S2**) confirms the formation of the α‐phase Ni(OH)_2_. A broad diffraction peak appears at 2θ of ∼11.35°, corresponding to d‐spacing of ∼7.8 Å​. The absence of any intense reflection at ∼19° indicates that the β‐Ni(OH)_2_ polymorph is not present [34]. Instead, broad peaks are observed at higher angles (e.g., ∼33.5°, 34.4°, and 38.8°) consistent with in‐plane (101), (012), and (015) reflections of α‐Ni(OH)_2_ [35, 36]. A relatively intense peak observed at ∼59.9° also corresponds to the (110) reflection of α‐Ni(OH)_2_. The peak broadening and low intensity (see Table S1 for peak assignments) suggest poor crystallinity and turbostratic disorder of the layers, as often seen in α‐Ni(OH)_2_​. Scanning electron microscopy (SEM) (Figure S3a) reveals that the α‐Ni(OH)_2_ material exhibits a three‐dimensional porous framework with open channels and voids. High‐magnification images confirmed the porous network morphology with interconnected nanosheets continuously distributed on the Ni foam substrate. This morphology would increase the accessible surface area and provide numerous active sites, while facilitating mass transport and gas diffusion during electrochemical reactions. High‐resolution transmission electron microscopy (HRTEM) further confirms the α‐Ni(OH)_2_ structure (Figure S3b) by clear lattice fringes with interplanar spacings of 0.268, 0.260, 0.232, and 0.154 nm, which match well with the d‐spacings calculated from the XRD pattern. The selected‐area electron diffraction (SAED) pattern (Figure S3c) consists of diffuse rings rather than sharp spots, indicating polycrystalline or turbostratically disordered structure. The principal SAED ring radii match the expected d‐spacings of α‐Ni(OH)_2_. The diffuse nature of the rings is consistent with random stacking and rotational misalignment of Ni(OH)_2_ layers​.

The Raman spectrum of the as‐synthesized sample (Figure 1d) further verifies the α‐phase structure and the presence of interlayer species. A prominent band observed at ∼471 cm^−1^ corresponds to the Ni–O lattice vibrational mode (bending mode, δ(Ni–O)) of α‐Ni(OH)_2_ [34]. In contrast, β‐Ni(OH)_2_ typically shows intense lattice mode near ∼318 and ∼449 cm^−1^​, so the ∼471 cm^−1^ feature is consistent with the α‐phase [37]. Additionally, a prominent peak at 1054 cm^−1^ is present, which is commonly attributed to interlayer NO_3_^−^ from the precursor [34, 38, 39]. An extended Raman analysis into the O–H stretching region (> 3000 cm^−1^) offered additional phase‐sensitive vibrational information [29]. As shown in Figure S4, distinct bands were observed at approximately 3560 and 3620 cm^−1^, corresponding to the stretching vibrations of lattice hydroxyl groups and interlayer water molecules, respectively [34]. The broadness of the O–H band in α‐Ni(OH)_2_ arises from the hydrogen‐bonded water and disorder in the interlayer, which contrasts with the more defined O–H vibration in β‐Ni(OH)_2_​.

The Raman spectra of the catalyst after electrochemical OER and UOR (Figure 1d) featured significant surface reconstructions of the α‐Ni(OH)_2_ into NiOOH during the anodic processes. For clarity, the post‐OER and post‐UOR samples refer to electrodes that underwent chronopotentiometry at 100 mA cm^−2^ for 1 h in 1 M KOH and in 1 M KOH + 0.33 M urea, respectively (Figure S1). In both cases, two dominant bands emerged at ∼470 and ∼550 cm^−1^. These bands are the signature vibrational modes of NiOOH, commonly assigned to the E_g_ bending mode (δ, ∼470 cm^−1^) and A_1g_ stretching mode (ν, ∼550 cm^−1^) of the Ni–O lattice in NiOOH​ [40]. The appearance of these peaks was accompanied by the concomitant decrease in the Ni(OH)_2_ band at ∼460 cm^−1^. Notably, the relative intensity of the δ‐mode compared to the ν‐mode (I_δ/ν_) has widely been used to distinguish β/γ‐NiOOH phases [41, 42]. Compared to the post‐OER sample, the post‐UOR sample displayed peaks at 471 cm^−1^ and 540 cm^−1^ with more prominent ν‐mode, indicating the formation of β‐NiOOH that is a thermodynamically more stable phase with less structural distortion. This phase selectivity is consistent with the electrochemical conditions: OER operates at higher anodic potentials (≥1.4 V vs. RHE) that promote the formation of oxidized Ni^4+^‐rich γ‐NiOOH, whereas UOR proceeds at lower potentials, favoring the stabilization of β‐NiOOH (Ni^3+^). These findings confirm that NiOOH phase evolution prefers γ‐NiOOH under OER, whereas β‐NiOOH was dominant after UOR [43].

Further insight into the surface chemical states was obtained by high‐resolution Ni 2p XPS, as shown in Figure 1e. In the α‐Ni(OH)_2_ sample, a small peak at ∼852.1 eV was identified and attributed to metallic Ni^0^ originating from the underlying Ni foam substrate. This Ni^0^ signal disappeared completely after OER, likely due to the higher anodic potential promoting full oxidation [44], whereas it remained partially observable after UOR, which occurs under milder conditions. The Ni 2p spectra also exhibit characteristic peaks for Ni^2+^ at 854.8 eV (2p_3/2_) and 872.5 eV (2p_1/2_), and for Ni^3+^ at 855.6 eV and 873.4 eV. Corresponding satellite peaks are observed at 860.7 eV and 878.8 eV, respectively [45, 46]. The resulting Ni^3+^/Ni^2+^ ratios were ∼1.98 after OER and ∼0.49 after UOR, indicating higher average Ni valency under OER (comparatively γ‐NiOOH‐like) and less oxidized surface under UOR (comparatively β‐NiOOH‐like). The O 1s spectra further revealed the distinct surface reconstruction during OER and UOR (Figure 1f). To ensure direct comparability across samples, we deconvoluted the O 1s spectra using a uniform four‐component model with fixed binding energies, namely 529.3 eV for M‐O, 530.1 eV for M‐OH, 530.7 eV for vacancy‐related (V_O_) oxygen, and 531.4 eV for molecular H_2_O [47, 48]. With this consistent scheme, the post‐OER electrode exhibits a pronounced increase in the V_O_, reflecting enhanced defect formation and a relatively γ‐NiOOH‐like surface. In contrast, the post‐UOR electrode shows stronger M‐O and less pronounced V_O_ features than post‐OER, consistent with a less defective, β‐NiOOH‐like surface. These O 1s trends are in accordance with the Ni 2p‐derived changes in Ni valency.

NiOOH exists in multiple structural phases, among which the β‐phase, composed primarily of Ni^3+^ centers, has been widely recognized as the catalytically active phase for the urea oxidation reaction (UOR) [49, 50, 51]. In alkaline media, Ni(OH)_2_ must first be electrochemically oxidized to NiOOH to enable urea oxidation, as Ni^2+^ alone lacks sufficient oxidative power to cleave the C–N and N–H bonds in urea [49]. This transition results in the formation of β‐NiOOH, where the higher oxidation state of nickel (Ni^3+^) facilitates multi‐electron transfer and acts as the true redox‐active center during UOR [49, 50]. Experimental studies have confirmed that urea oxidation does not proceed on Ni(OH)_2_ surfaces until they are oxidized to NiOOH, and that Ni^3+^‐rich or structurally disordered oxyhydroxide phases exhibit lower onset potentials and higher catalytic activity [50, 51]. These findings highlight the essential role of Ni^3+^ in β‐NiOOH for enabling the energetically demanding steps of urea oxidation and are consistent with the behavior of Ni‐based catalysts in other oxidative processes such as the OER. Hence, establishing and maintaining Ni^3+^ centers in β‐NiOOH is a prerequisite for efficient UOR catalysis. Our Raman spectra in Figure 1d also demonstrated the formation of β‐NiOOH under UOR potential conditions, consistent with the previous studies [2]. Based on the experimental observations, DFT calculations were performed with information of the β‐NiOOH phase and its Ni^3+^ active sites.

However, the precise atomic structure of β‐NiOOH, especially the distribution of hydrogen atoms, is still not fully resolved, making it difficult to fully comprehend its electrocatalytic behavior [52]. Theoretical studies have proposed various structures with different Ni oxidation states and hydrogen distributions, highlighting the importance of resolving the detailed atomic arrangement of β‐NiOOH [24]. To verify that Ni atoms in our model retain the Ni^3+^ oxidation state, we performed spin‐polarized DFT calculations and analyzed the local magnetic moments on the β‐NiOOH (001) surface. Each Ni atom exhibits a computed magnetic moment of approximately 1.23 μ_B_, corresponding to one unpaired electron as expected for a 3d^7^ configuration in an octahedral crystal field. The above result confirms that our β‐NiOOH model reliably maintains Ni in the +3 oxidation state, as intended [53].

The V_O_ sites could be critical active sites in UOR mechanisms, significantly influencing adsorption processes, electrocatalytic efficiency, and selectivity, which are often overlooked in previous studies. Among the limited literature addressing V_O_, Tatarchuk et al. reported that urea coordination is more favorable on surfaces with higher concentrations of vacancies, correlating with increased UOR activity in disordered materials. Furthermore, they asserted that a lack of vacancies favored C‐N cleavage, resulting in NO*_x_* ^−^ and OCN^−^ formation, while the abundance of vacancies suppressed C‐N cleavage, favoring N_2_ and CO_2_ formation pathway [25]. However, their study exhibits several limitations; for example, the hydrogen distribution on the NiOOH surface was not adequately modeled, leading to uneven charge distribution on Ni. In addition, their calculations focused primarily on a well‐known dual N adsorption configuration for urea, neglecting the exploration of alternative adsorption structures such as single O adsorption to the V_O_ site. Moreover, the reaction pathways were oversimplified, constraining the accuracy to align computational predictions with experimental results. While highlighting the roles of V_O_ in UOR, a more comprehensive approach was necessary by incorporating accurate surface modeling and diverse adsorption configurations to fully elucidate the UOR mechanism.

To this end, we focus on the hydrogen distribution in β‐NiOOH that maintains the Ni oxidation state as Ni^3+^, a key factor for preserving its electronic properties during UOR. Using the established bulk crystal structure, we constructed a slab surface containing 32 nickel atoms. Among the two potential slab terminations, we selected β‐NiOOH (001) with the lowest surface energy for further simulations (Figure 2a). Detailed information on bulk structure and surface terminations can be found in Supporting Information (Figure S5). To unravel the interplay of oxygen‐related vacancies in NiOOH during urea electrooxidation, the formation energies of both oxygen and hydroxide vacancies on NiOOH (001) were calculated. Although hydroxide vacancies are less likely to form under alkaline conditions, they were also included in the analysis for a comprehensive understanding of potential vacancy sites and their impact on catalytic behavior. As expected, the results revealed that hydroxide vacancy formation was not favored with a vacancy formation energy of 0.53 eV, while V_O_ was thermodynamically favored with a vacancy formation energy of ‐1.08 eV (Figure 2b,c). This indicates that V_O_ formation is inherently more likely to occur under experimental conditions, in line with the O 1s spectra (Figure 1f), significantly influencing the surface chemistry and catalytic behavior of NiOOH. These findings underscore the critical roles of V_O_ as active sites in facilitating UOR.

**FIGURE 2:**
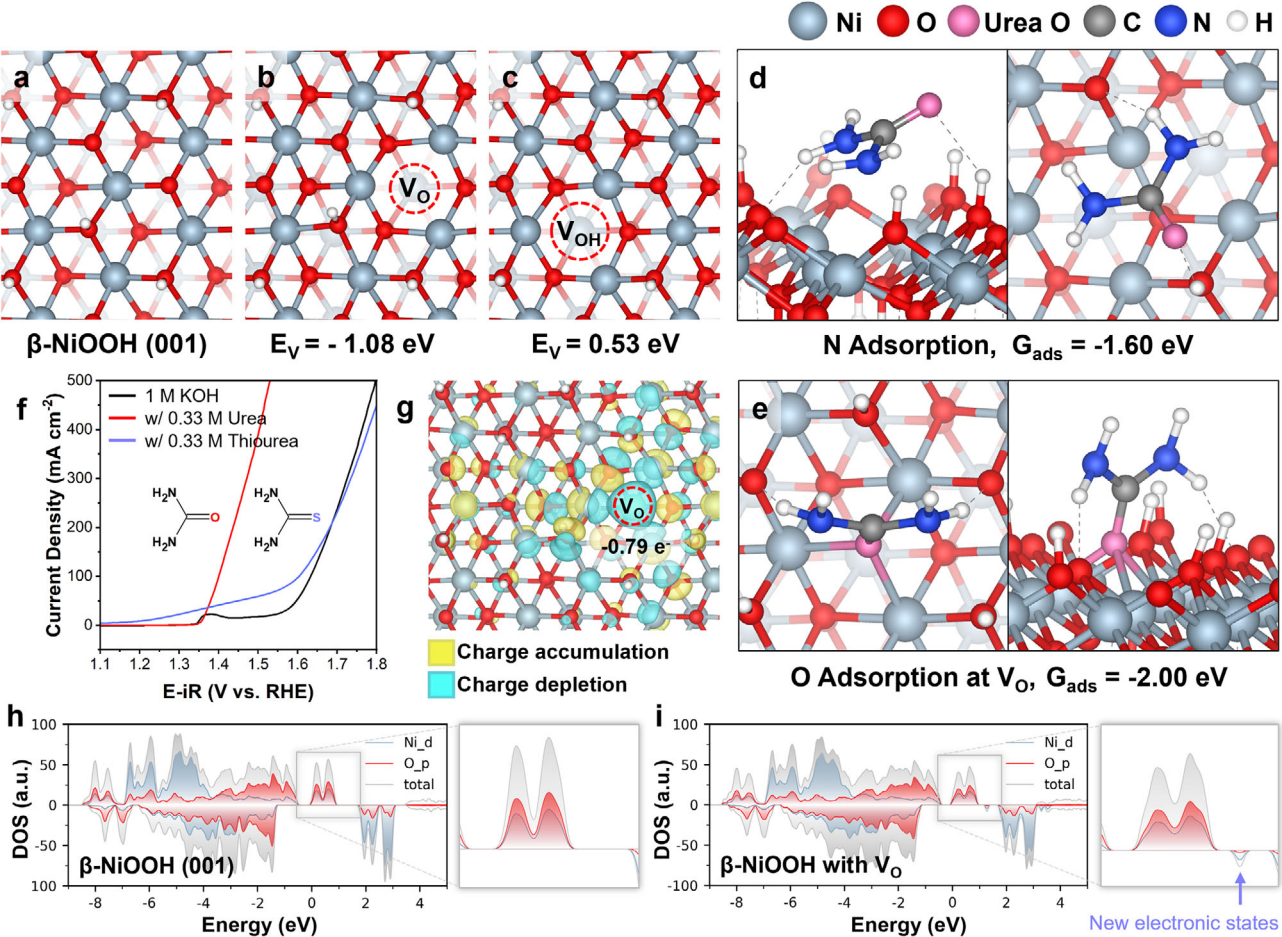
(a) DFT‐optimized β‐NiOOH (001) surface structure with (b) oxygen vacancy and (c) hydroxide vacancy with vacancy formation energies. Top and side view of the optimized urea structures for (d) N adsorption (−1.60 eV) and (e) O adsorption (−2.00 eV) configuration with adsorption energies. (f) LSV curve comparison of the NiOOH electrode was conducted in a 1 M KOH solution containing 0.33 M thiourea and urea. With thiourea, current activity is very small compared to urea, indicating oxygen vacancy presence and single O adsorption configuration at the vacancy site. (g) Charge density difference plot with Bader charge analysis for oxygen vacancy formation (h) Partial density of states of β‐NiOOH (001) surface and (i) β‐NiOOH (001) surface with oxygen vacancy.

Notably, the V_O_ sites serve as potential sites for urea adsorption, dictating the intricacies of the electrocatalytic process. In order to explore the most optimized adsorption configuration of urea, different adsorption configurations were tested, and the following results were comparatively analyzed. As previously reported, urea typically adsorbs onto metal oxide catalysts via a dual N adsorption configuration, wherein both N atoms of the urea molecule interact simultaneously with metal sites. Nevertheless, when a surface such as metal oxide is saturated with hydroxide and oxygen, it is challenging for urea to find a vacant valence electron site suitable for adsorption. Under such conditions, urea is more likely to adhere to the surface through hydrogen bonding. Consistent with these expectations, the dual N adsorption configuration was obtained, where the oxygen atom of urea interacts with a hydrogen atom (*H) on the surface, while the hydrogen atom of urea interacts with a surface oxygen atom (*O) via hydrogen bonding. This configuration exhibited an adsorption Gibbs free energy of −1.60 eV (Figure 2d). On the other hand, on surfaces with thermodynamically favored V_O_, a single O adsorption was found to be the most stable configuration, where the oxygen atom of urea fills the V_O_ (Figure 2e) with an adsorption Gibbs free energy of −2.00 eV (0.40 eV lower than the nitrogen‐based adsorption). Notably, no direct interaction between the metal site and N atoms of urea was observed.

In order to experimentally validate the occurrence of V_O_ and urea‐O adsorption, we utilized thiourea (SC(NH_2_)_2_) as an analog (oxygen atom substituted with sulfur atom). Unlike the UOR kinetics, which elicited a distinct anodic current at ∼1.36 V (Figure 2f), the thiourea oxidation was negligible according to the anodic response. The small currents from ∼ 1.1 V RHE in the presence of thiourea were clarified by additional experiments (Figure S6), which revealed a gradual decrease in the current density and visible discoloration of the electrolyte during consecutive LSV scans. These results suggest that the observed current was ascribed to Ni dissolution induced by corrosive sulfur‐containing oxidants (S_2_O_4_^2−^, SO_2_^−^) due to thiourea decomposition in the presence of dissolved oxygen [54, 55, 56]. Thus, the urea adsorption would occur specifically via its oxygen atom at V_O_ sites of catalysts, a process that thiourea cannot replicate due to the presence of sulfur instead of oxygen. These experimental results provide strong evidence for the critical role of V_O_ in UOR. Furthermore, the observed behavior aligns closely with theoretical predictions, reinforcing the validity of the proposed UOR mechanism and highlighting the importance of V_O_ sites to enhance the UOR efficiency.

To investigate the impact of V_O_ in NiOOH (001) at the atomic scale, an in‐depth charge density analysis was performed (Figure 2g). Upon the formation of V_O_, 0.79 e is depleted at the site, resulting in an accumulation of electrons around the adjacent Ni and O atoms, indicating charge redistribution on the surface. In addition, charge density difference analysis confirms that Ni gained 0.32 e when the V_O_ occurred. This redistribution is crucial, as it further reduces the surface charge density and facilitates the adsorption of urea at sites with the charge density deficit. In addition, to assess the effect of V_O_ formation on electronic states, partial density of states (PDOS) calculations were carried out (Figure 2h,i). The formation of V_O_ sites introduces new electronic states that narrow the original gaps, enhancing conductivity, which promotes easier adsorption and subsequent oxidation of adsorbates like urea. Based on the charge density analysis and PDOS results, the electronic environment modified by the V_O_ is likely to play a key role in modulating the catalyst's adsorption properties, ultimately influencing the overall catalytic process. This finding highlights the critical importance of electronic structure evaluation in designing and optimizing catalytic systems, particularly in the context of urea adsorption and reaction pathways in NiOOH‐based catalysts.

In order to evaluate the impact of O vacancy on Ni oxidation state, further electronic structure analysis was performed. Upon the removal of a lattice oxygen atom from NiOOH, the local spin and charge equilibrium are perturbed, leading to the redistribution of electronic density previously associated with the O^2−^ anion. This redistribution results in increased electron density around neighboring Ni sites, partially reducing them from the Ni^3+^ state toward a Ni^2+^‐like configuration. Charge density difference analysis (Figure 2g) confirms this behavior, showing the accumulation of electron density around Ni atoms adjacent to the vacancy. Bader charge analysis further supports this interpretation. The three Ni atoms nearest the vacancy exhibit an increase in valence electron count from an average of 8.13 e (pristine surface) to 8.72 e (defective surface), reflecting a gain of ∼0.6 e per Ni atom. The total electron gain of 0.79 e (when calculated for all Ni atoms in the system) suggests that the electron redistribution is spread across the entire Ni site. This trend is consistent with a shift in formal oxidation state toward Ni^2+^. Spin‐polarized DFT calculations were also conducted to evaluate the magnetic properties associated with this local reduction. The average magnetic moment of the three nearby Ni atoms increases from 1.22 to 1.52 μ_B_, indicating enhanced spin polarization. These values are consistent with prior studies: magnetic moments of ∼1.0 μ_B_ are typically associated with Ni^3+^ (3d^7^, one unpaired electron), while values of ∼1.7–1.8 μ_B_ are characteristic of Ni^2+^ (3d^8^, two unpaired electrons), and near‐zero values correspond to low‐spin Ni^4+^ (3d^6^) [53]. The increase to 1.52 μ_B_ supports the interpretation that the Ni atoms near the vacancy are partially reduced toward the Ni^2+^ state, while also exhibiting the expected degree of delocalization and covalent character inherent to β‐NiOOH. From a catalytic standpoint, this local electronic restructuring is highly relevant, as the oxidation state of Ni modulates its affinity for the key oxygenated intermediates. Moreover, the oxygen vacancy itself introduces a new active site that may facilitate the adsorption of urea or other species. These findings highlight the dynamic nature of the Ni oxidation state in defective NiOOH, underscoring the importance of electronic structure analysis in understanding and optimizing catalytic behavior.

The single O adsorption configuration of urea plays a critical role in elucidating the complex UOR mechanism, distinguishing it from previously reported dual N adsorption configurations [14, 57]. When urea is anchored to the V_O_ on the catalyst surface, it adopts a stable adsorption mode resembling a socket‐like attachment. In this configuration, the hydrogen atoms in the NH_2_ groups of urea are easily detached due to strong hydrogen bonding interactions with neighboring oxygen atoms on the catalyst surface or in the solvent. This facilitates dehydrogenation and subsequent oxidation steps. In contrast, the dual N adsorption configuration, where both nitrogen atoms of urea are bound to the catalyst surface, is less stable. The more destabilized adsorption in this configuration hinders the oxidation process by limiting effective interactions of urea (and oxidation intermediates) with the catalyst. This shift in understanding, derived from insights into the oxidation state of Ni and the role of V_O_ sites, highlights the importance of interaction between urea and active NiOOH surfaces. These findings also lay a solid foundation for optimizing electrocatalyst designs tailored for UOR.

Based on the preference of urea for single O adsorption at oxygen vacancy sites, the UOR pathways were designed and evaluated. Previous studies have predominantly focused on the dehydrogenation of NH_2_ species or C‐N bond cleavage, often linked with dual N adsorption [2, 25, 57]. On the other hand, in this study, a comparative Gibbs free energy reaction diagram analysis of these processes was conducted, with an emphasis on single O adsorption at V_O_ sites. Using the single‐O adsorption configuration, urea can follow two distinct oxidation pathways: (a) a pathway involving complete dehydrogenation, leading to the formation of N_2_ and CO_2_, or (b) a pathway involving a combination of dehydrogenation and C‐N cleavage steps, resulting in the formation of OCN^−^ and NH_2_ species adsorbed onto nearby lattice oxygen, which is subsequently oxidized to NO_2_^−^ (Figure 3a,b). When designing overall UOR pathways, dehydrogenation and C‐N cleavage subsequent to the urea adsorption must be carefully considered. During dehydrogenation, the two N atoms in urea remain closely connected through the central carbon atom, facilitating the formation of two‐nitrogen species that can ultimately bond to form N_2_. In contrast, C‐N cleavage results in the separation of the two nitrogen atoms, leading to single N‐containing products such as OCN^−^ and NO_2_^−^. While N_2_ and CO_2_ have conventionally been regarded as the primary products of the UOR, we seek to re‐evaluate the traditional mechanism in the context of urea‐oxygen adsorption.

**FIGURE 3:**
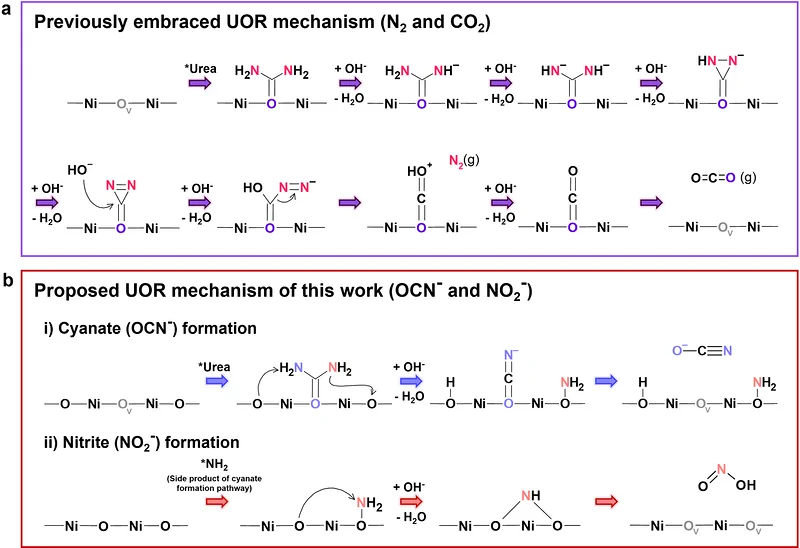
Urea oxidation reaction mechanism: (a) previously embraced UOR mechanism for N_2_ and CO_2_ formation and (b) proposed UOR mechanism of this work for OCN^−^ and NO_2_^−^ formation on NiOOH (001) surface with oxygen vacancy.

Using the single O adsorption configuration, we revisited the energetics of the N_2_ and CO_2_ formation pathway. In this pathway, complete dehydrogenation steps are followed by C‐N cleavage, enabling the release of N_2_ and CO_2_ as final products. The proposed pathway involves the following sequence of intermediates: * → *OC(NH_2_)_2_ → *OCNH·NH_2_ → *OCNHNH → *OCNNH → *OCN_2_ → *OCOH + N_2_ → *OCO → CO_2_. For the formation of OCN^−^ and NO_2_^−^, three distinct pathways were evaluated: (1) C‐N cleavage, (2) dehydrogenation, and (3) a combination of C‐N cleavage and dehydrogenation. Among these, the combination pathway was found to be the most energetically favorable. The proposed OCN^−^ formation pathway on NiOOH proceeds through a series of intermediates: * → *OC(NH_2_)_2_ → OCN/*H/*NH_2_. Subsequently, the formation of NO_2_^−^ occurs as a continuation of this pathway. During the OCN^−^ formation, *NH_2_ is generated as a key intermediate, which interacts with lattice oxygen (*2O) to form *H_2_NO_2_. This intermediate then undergoes further oxidation to produce HNO_2_, which is eventually converted to NO_2_^−^ in the electrolyte. These re‐evaluations allow for a direct comparison with the mechanisms, highlighting the role of single O adsorption in determining the energetics and feasibility of various reaction pathways.

To comprehensively analyze the energetics of UOR pathways, all intermediates were calculated in terms of Gibbs free energy, and reaction profiles were constructed. This study extends beyond the conventional comparison of the two predominant pathways in urea oxidation—dehydrogenation and C‐N bond cleavage—by evaluating a hybrid pathway that integrates both processes. Initially, the dehydrogenation and C‐N cleavage pathways were assessed independently. The first dehydrogenation step, converting *OC(NH_2_)_2_ to *OCNHNH_2_, required a Gibbs free energy change of 1.54 eV, while the C‐N cleavage step from *OC(NH_2_)_2_ to *OCNH_2_ exhibited that of 1.95 eV, indicating that dehydrogenation is thermodynamically more favorable than direct C‐N bond cleavage (Figure 4a). These results suggest that dehydrogenation of the NH_2_ groups in urea is preferred over C‐N cleavage of urea on the β‐NiOOH catalyst. A hybrid pathway combining dehydrogenation followed by C‐N cleavage was evaluated, reflecting a deliberate effort to explore more complex and realistic reaction mechanisms. Unlike traditional approaches that often focus on single‐step or isolated pathways, this study aimed to investigate the interplay and synergies between multiple reaction pathways to uncover the energetically and mechanistically most favorable sequence. By combining dehydrogenation with C‐N bond cleavage, occurring in two distinct yet stoichiometrically equivalent configurations, this study provides deeper insights into the transient interactions and adsorptive behaviors on the catalyst surface. Initially, one NH_2_ group undergoes dehydrogenation to form NH. Simultaneously, the second NH_2_ group undergoes C‐N bond cleavage, with an associated Gibbs free energy change of 1.49 eV. This sequence is facilitated by a transient interaction between an N atom from urea and an adjacent O atom on the catalyst surface. Comparative analysis reveals that the hybrid pathway, incorporating both dehydrogenation and C‐N cleavage, is energetically the most favorable, with a Gibbs free energy change of 1.49 eV (slightly lower than the 1.54 eV required for dehydrogenation alone). This small energy difference of 0.05 eV indicates a preference for OCN^−^ formation alongside the adsorption of NH_2_ species onto adjacent lattice oxygen atoms on the NiOOH surface. Also, for the OCN^−^ formation pathway, the combination of the dehydrogenation and C‐N cleavage steps would be the PDS.

**FIGURE 4:**
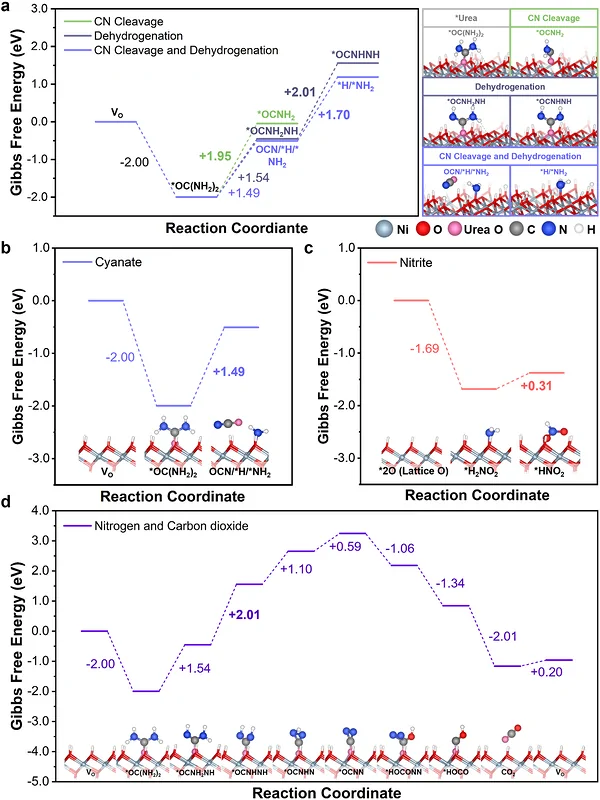
Gibbs free energy diagrams of (a) comparison of C‐N Cleavage, Dehydrogenation, and a combination of C‐N Cleavage and dehydrogenation pathways, (b) OCN^−^ formation pathway, (c) NO_2_^−^ formation pathway, and (d) N_2_ and CO_2_ formation pathway.

Moreover, the OER was also evaluated at the oxygen vacancy site of β‐NiOOH (001) for comparison (Figure S9). The PDS for OER, the desorption of O_2_ (*OOH → O_2_), exhibited a Gibbs free energy barrier of 3.25 eV, corresponding to an overpotential of η = 2.02 V. This value is 1.76 eV higher than the PDS for OCN^−^ and 2.94 eV higher than the PDS for NO_2_^−^ (Figure S10), confirming that the pathways for OCN^−^ and NO_2_^−^ formation are energetically favorable. As OCN^−^ forms and is released, the NH_2_ species adsorbs onto an adjacent lattice oxygen atom (Figure 4b). The subsequent desorption of HNO_2_, with a Gibbs free energy change of 0.31 eV, represents the PDS in the NO_2_^−^ formation pathway (Figure 4c). The HNO_2_, a weak acid with a pK_a_ of 3.29, should be eventually converted to NO_2_^−^ in the electrolyte [58, 59]. Comparatively, the energy barriers for the OCN^−^ and NO_2_^−^ pathways are significantly lower than for the N_2_/CO_2_ pathway, which was re‐evaluated based on single O adsorption of urea. Even when N_2_ and CO_2_ were the primary products, the previously reported PDS of CO_2_ detachment from the surface was not observed in this study (Figure 4d). All DFT optimized intermediate configurations can be found in SI (Figures S7 and S8). These findings emphasize the thermodynamic favorability of OCN^−^ and NO_2_^−^ formation under UOR conditions and provide a refined understanding of UOR mechanisms on NiOOH.

To validate the proposed mechanism from the theoretical predictions, we conducted an extensive experimental study to identify the actual UOR products by ion chromatography (IC). Mixed standards of NO_2_^−^, OCN^−^, and NO_3_^−^ were prepared at 0.2, 0.4, 0.8, and 1.6 mM. The post‐electrolysis electrolyte (1.55 V, 5 h in 1 M KOH + 0.33 M urea) was diluted 100‐fold and analyzed together with the standards. The sample exhibited peaks at the retention time for NO_2_^−^ and OCN^−^, while NO_3_^−^ was below the detection limit (Figure S11). Quantification against the standards yielded [NO_2_^−^] = 137 mM and [OCN^−^] = 112 mM in the undiluted electrolyte (Figure 5a). Based on these concentrations, together with the total passed charge (3409.65 C) and the final electrolyte volume (35 mL), the Faradaic efficiencies (FEs) were estimated to be FE(NO_2_^−^) = 67.8%, FE(OCN^−^) = 11.1%, and FE(N_2_) = 21.4%, closing the charge balance at ∼ 100.3%. Note that the N_2_ generation was based on a nitrogen mass balance ([N_2_] ≈ 36.0 mM) considering the reduced [urea]. Although the NO_2_^−^ to OCN^−^ molar ratio deviates from 1:1, likely due to concurrent homogeneous chemistry in alkaline solution, the predominance of NO_2_^−^ and OCN^−^ together with the low OER current at 1.6 V in the urea‐free electrolyte (Figure 1a) indicates that the N‐products from the UOR account for essentially all of the anodic charge. It supports that the DFT‐derived OCN^−^/NO_2_^−^ pathway (Figure 3b) dominates the previously embraced N_2_/CO_2_ pathway.

**FIGURE 5:**
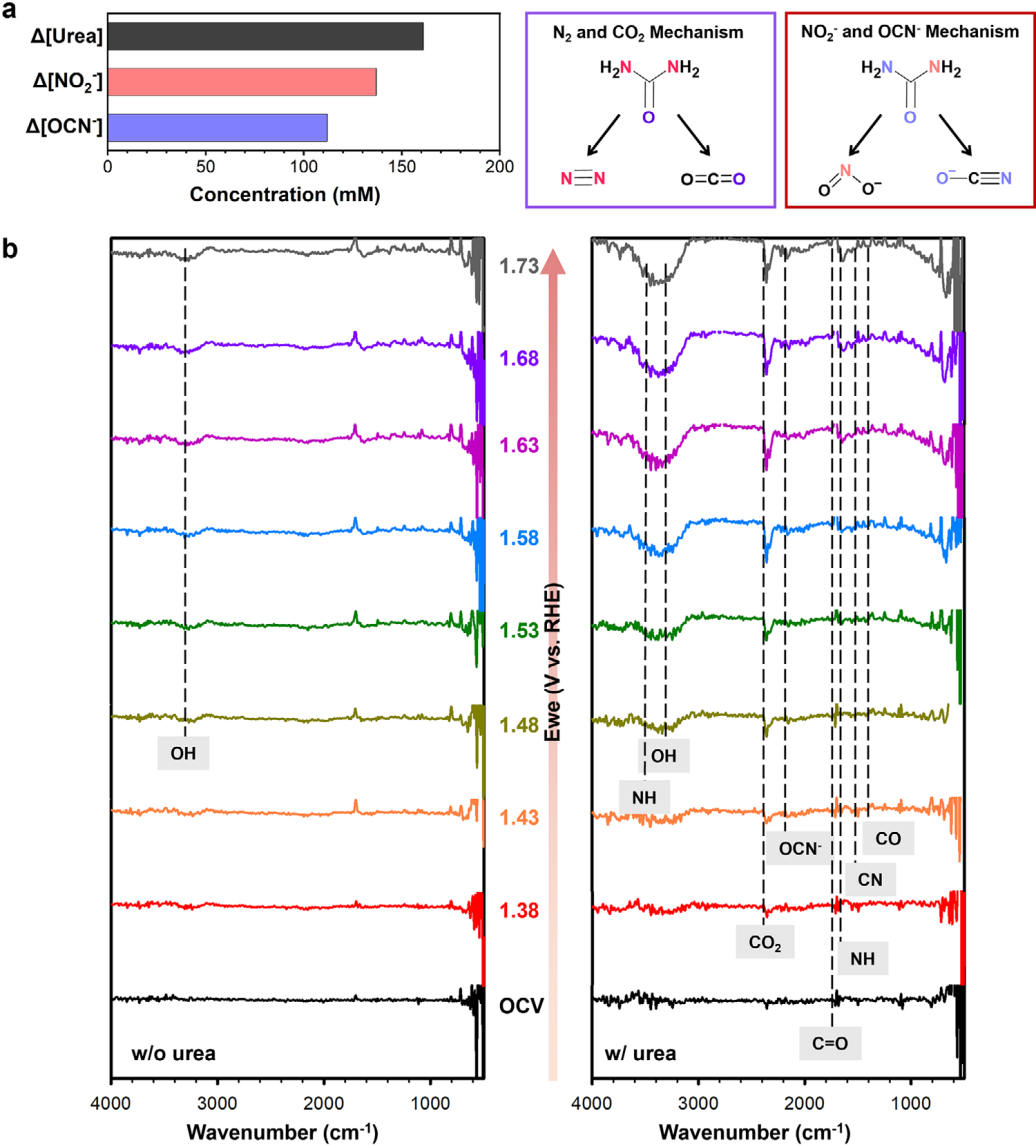
(a) Quantification of urea, nitrite, and cyanate after electrolysis of 0.33 M urea in 1 M KOH at 1.55 V versus RHE for 5 h. (b) In situ FTIR spectra of α‐Ni(OH)_2_ in 1 M KOH electrolyte without (left) and with 0.33 M urea (right), obtained at various potentials from OCV to 1.73 V versus RHE.

To further validate the UOR pathways toward OCN^−^ and NO_2_^−^, in situ FT‐IR analysis was performed to identify adsorbed intermediates during the reaction. For accurate observation, differential spectra were displayed for each applied potential compared to the signals at OCV (open circuit voltage). As shown in Figure 5b, in the absence of urea, no characteristic peaks were observed except for the broad O‐H stretching (ν) band at 3230 cm^−1^, which is attributed to surface OH/H‐bonded water on NiOOH [60]. In contrast, in urea‐containing solutions, a clear potential‐dependent band appears at 3420 cm^−1^, assigned to the ν(N‐H) vibration of adsorbed *NH/*NH_2_ species, highlighting *NH as a key intermediate [61]. By comparison, features near ∼2180 cm^−1^ from asymmetric stretching of OCN (ν_as_(OCN), OCN^−^/*OCN) and ∼1480 cm^−1^ (ν(C‐N), urea‐derived adsorbates) were very weak under our conditions. Their low intensities are consistent with rapid desorption of *OCN to solution as OCN^−^ to lower the surface coverage [61]. In addition, bands appeared at 1720 cm^−1^ and 2390 cm^−1^ indicated CO_2_ formation; the 1720 cm^−1^ feature was ascribed to carbonate (CO_3_^2−^) formed by CO_2_ hydration in alkaline media, while the 2390 cm^−1^ band corresponds to the ν_as_ of dissolved CO_2_ [48]. These weak signals, together with the product analysis, indicate that CO_2_‐related pathways are minor compared with the dominant OCN^−^/NO_2_^−^ pathways, in agreement with the DFT‐predicted selectivity and Faradaic‐efficiency. Accordingly, the reactions can be summarized as follows:

Overall urea oxidation reaction:

(2) $$\begin{array}{cc} & \mathrm{NiOOH}*\mathrm{OC}{\left({\mathrm{NH}}_{2}\right)}_{2}+{\mathrm{OH}}^{-}\to \mathrm{OC}N^{-}+\mathrm{HN}O_{2}+{\mathrm{Ni}}^{2+}\\ & \;+\;H_{2}O\;+3H^{+}+5e^{-} \end{array}$$

Urea oxidation reaction pathway consists of two steps as seen below.

Step1: Partial oxidation of urea to OCN^−^

(3) $$\begin{array}{cc} & \mathrm{NiOOH}*\mathrm{OC}{\left(NH_{2}\right)}_{2}+OH^{-}\to \mathrm{OC}N^{-}+\mathrm{NiOOH}*NH_{2}\\ & +H_{2}O\;+H^{+}+e^{-}\; \end{array}$$

Step2: Successive oxidation of *NH_2_ (byproduct of urea oxidation) to HNO_2_

(4) $$\mathrm{NiOOH}*NH_{2}\to \mathrm{NiOOH}*\mathrm{NH}+H^{+}+e^{-}\;$$

(5) $$\mathrm{NiOOH}*\mathrm{NH}\to \mathrm{NiOOH}*N+H^{+}+e^{-}\;$$

(6) $$\mathrm{NiOOH}*N\to Ni^{2+}+\mathrm{HN}O_{2}+2e^{-}$$

When HNO_2_ detaches from the NiOOH surface to the solution, HNO_2_ will rapidly deprotonate:

(7) $$\mathrm{HN}O_{2}⇌H^{+}+{\mathrm{NO}}_{2}^{-}\;$$

Following its formation on the surface, HNO_2_ is readily deprotonated in the alkaline electrolyte to form NO_2_^−^, the thermodynamically stable and experimentally observed product. This rapid sequence of oxidation and desorption steps contributes to the difficulty in detecting intermediate species, as many quickly dissolve into the electrolyte upon formation.

Nevertheless, the in situ FTIR results are consistent with the predicted pathway, supporting the hypothesis that OCN^−^ formation proceeds via a low‐intermediate‐coverage mechanism. Equations (2) and (3) represent the core UOR process on β‐NiOOH, where OCN^−^ and NO_2_^−^ emerge as the dominant products. Importantly, the oxygen vacancy (V_O_) is confirmed as the active site facilitating these transformations, consistent with both theoretical modeling and experimental observations. By integrating computational and spectroscopic evidence, this work advances the mechanistic understanding of UOR on NiOOH (001). The identification of OCN^−^ and NO_2_^−^ as primary products—contrary to the traditionally expected N_2_ and CO_2_—represents a paradigm shift in UOR research, offering new insights for designing more selective and efficient Ni‐based electrocatalysts.

## 3 Conclusion

This study presents both experimental and computational analyses that provide a deeper understanding of the UOR mechanism on NiOOH catalysts. Experimentally, significant UOR current densities of 100 mA cm^−2^ and 500 mA cm^−2^ were achieved at potentials of 1.40 V RHE and 1.53 V RHE, respectively, markedly outperforming the OER, which required 1.79 V RHE to achieve 500 mA cm^−2^. IC and in situ FT‐IR analyses confirmed the presence of OCN^−^ and NO_2_^−^ as detectable products, substantiating the mechanistic predictions and highlighting the efficiency of UOR as a competitive anodic reaction. Complementing these experimental results, the computational investigations revealed critical insights into the UOR mechanism on β‐NiOOH (001). Oxygen vacancies were identified as energetically favorable (−1.08 eV) and functioned as key active sites, significantly influencing the surface chemistry of β‐NiOOH (001). Urea adsorption at these vacancies in a single O adsorption configuration was identified as the most stable state, providing a foundation for subsequent mechanistic exploration. Comparative evaluations of reaction pathways indicated that a hybrid mechanism, incorporating both dehydrogenation and C–N cleavage steps, is energetically preferred. Mechanistic modeling further elucidated that while the dehydrogenation pathway produces N_2_ and CO_2_ as terminal products, the hybrid pathway generates OCN^−^ and *NH_2_ intermediates. The latter species, *NH_2_, undergo oxidation via lattice oxygens to form HNO_2_, which is subsequently converted into NO_2_^−^. The novelty of our work lies in the clarification of urea oxidation reaction mechanism. Although OCN^−^ and NO_2_^−^ were often identified as products of UOR, their detailed formation pathways were unclear and traditionally assumed formation of N_2_ and CO_2_ were often questioned. Moreover, the proposed hybrid mechanism demonstrated a significantly lower energy barrier (1.49 eV) compared to that of OER (3.25 eV), emphasizing UOR as a more energy‐efficient anodic process. These findings mark a paradigm shift in the understanding of urea electrooxidation, presenting UOR as a promising alternative for hydrogen production while leveraging waste urea as a resource. By systematically demonstrating the impact of oxygen vacancies on nickel oxidation states and urea adsorption configurations, this study underscores the importance of tailored catalyst design for enhancing UOR performance. This work also addresses the mechanistic ambiguities in UOR and establishes a foundation for designing advanced catalysts with improved efficiency and selectivity by integrating experimental observations and theoretical insights. This approach not only overcomes the limitations of conventional NiOOH catalysts but also provides a roadmap for optimizing UOR processes in hydrogen production and environmental remediation.

## Conflicts of Interest

The authors declare no conflicts of interest.

## Supporting information




**Supporting File 1**: exp270189‐sup‐0001‐SuppMat.pdf.

## Data Availability

The data that support the findings of this study are available from the corresponding author upon reasonable request.
