# Supplementary material for: Mechanistic Understanding of Nickel Catalyzed Urea Oxidation Reaction to Cyanate and Nitrite
Source: Exploration (Beijing). 2026 Jun 5;6(5):20240378. doi: 10.1002/EXP.20240378 (PMC13394143; doi:10.1002/EXP.20240378)
Supplement: Supplementary file 1 [file EXP2-6-20240378-s001.pdf]

Supporting Information

Mechanistic Understanding of Nickel Catalyzed Urea Oxidation Reaction to Cyanate and Nitrite

Kyu In Shim‡,a, Jiseon Kim‡,b, Miyeon Kim a, Kangwoo Chob,, and Jeong Woo Hana,**

[a] Department of Materials Science and Engineering, Research Institute of Advanced Materials, Seoul National University, Seoul 08826, Republic of Korea
[b] Division of Environmental Science and Engineering, Pohang University of Science and Technology (POSTECH), Pohang 37673, Republic of Korea

E−mail: kwcho1982@postech.ac.kr, jwhan98@snu.ac.kr

‡These authors equally contributed to this work.

Computational Details

All Density Functional Theory (DFT) calculations were performed using the Vienna Ab Initio Simulation Package (VASP) with the projector augmented wave (PAW) method.[1] The exchange-correlation energy was treated within the framework of the Bayesian error estimation functional with van der Waals correlation (BEEF-vdW).[2] A plane-wave basis set with a cutoff energy of 400 eV was employed to expand the wave functions. [3] A 4 × 4 × 4 and 2 × 2 × 1 Monkhorst-Pack *k*-point mesh was used for Brillouin zone sampling for bulk and surface, respectively. Structural optimizations were conducted until the forces on each atom were less than 0.03 eV/Å.[4] The electronic structure calculations were performed with spin polarization, considering various magnetic configurations. The convergence criteria for geometry optimizations and energy calculations were set to 1.0×10^{-4} eV, respectively.[5] The adsorption energies were obtained by considering a vacuum thickness greater than 15 Å to minimize spurious interactions between periodic images.

Hubbard U Correction

The DFT calculations included the implementation of the Hubbard *U* correction to correct the self-interaction error and address on-site Coulomb repulsion in transition metal oxides as seen

below:

$$E_{total} = E_{DFT} + \frac{U-J}{2}\sum_{\sigma} n_{m,\sigma} - n_{m,\sigma}^2,$$

where E_{total} is the total energy, E_{DFT} is the calculated total energy, U is the on-site Coulomb repulsion, J is the exchange interaction, n is the atomic orbital occupation number.[6] The exchange interaction can be incorporated into the Coulomb term, where the effective Hubbard U as

$$U_{eff} = U - J$$

Specifically, U_{3d} value for Ni was assigned as 5.5 eV, crucial for accurately capturing electronic correlations. This DFT+U methodology, executed using the Vienna Ab Initio Simulation Package (VASP), utilized a portion of U as the exchange parameter J. The systematic adjustment of U values offered valuable insights into how electron correlations influence both electronic structure and catalytic properties.

Vacancy Formation Energy

The presence of vacancies in the crystal lattice of catalyst materials can significantly impact their catalytic activity. To evaluate the formation of oxygen vacancy, oxygen vacancy formation energy was calculated using below equation:

$$E_v = E_{surface\ with\ O\ vacancy} - E_{surface} + \frac{1}{2}\mu_{O_2},$$

where is $E_{surface\ with\ O\ vacancy}$ is the total energy of surface model with one oxygen vacancy, $E_{surface}$ is the total energy of surface model, and μ_{O_2} is the chemical potential of oxygen gas. The chemical potential μ_{O_2} is determined as the energy of an isolated oxygen gas.

To evaluate the formation of hydroxide vacancy, hydroxide vacancy formation energy was calculated using below equation:

$$E_v = E_{surface\ with\ OH\ vacancy} - E_{surface} + \mu_{OH},$$

where is $E_{surface\ with\ OH\ vacancy}$ is the total energy of surface model with one hydroxide vacancy, $E_{surface}$ is the total energy of surface model, and μ_{OH} is the chemical potential of hydroxide.

$$\mu_{OH} = \mu_{H_2O} - \frac{1}{2}\mu_{H_2},$$

The chemical potential μ_{OH} is determined by the chemical potential of H_2O gas subtracted by half of the chemical potential of H_2. The chemical potentials are determined as the energy of an isolated H_2O and H_2 gas.

Adsorption Energy

The more negative value of oxygen adsorption energy indicates stronger binding of oxygen-related compounds to the catalyst surface. Moreover, it indirectly signifies lower electrochemical impedance, indicating that it is easier to transfer electrons and ions at the interface between the electrode and the electrolyte. Therefore, the adsorption energy (E_{ads}) of oxygen can be utilized as a critical tool to assess electrochemical impedance using below equation:

$$E_{ads} = E_{total} - E_{surface} - E_{adsorbate},$$

where E_{ads} is the total energy of system with adsorbed species, $E_{surface}$ is the total energy of the bare surface, and $E_{adsorbate}$ is the total energy of the gas phase adsorbate.

Surface Energy

The surface energy (γ) was calculated using the formula:

$$\gamma = \frac{1}{2A} (E_{slab} - nE_{bulk})$$

Here, E_{slab} is the total energy, is the E_{bulk} is the total energy of the bulk material, n is the stoichiometry parameter, and A is the surface area of the slab.

Gibbs Free Energy

The Gibbs free energy with thermal and entropic correction was employed using VASPKIT python module[7] and below equation:

$$G(T,P) = U(T) + \varepsilon_{ZPE} + PV - TS(T),$$

where U(T) and S(T) are the internal energy and entropy, which take electronic, translational, rotational, and vibrational energy contributions depending on the temperature into account. P, V, and T are the pressure, volume, and temperature, respectively. ε_{ZPE} is the zero-point energy.

The Gibbs free energy (ΔG) upon the adsorption of intermediates was calculated using the equation below:

$$\Delta G = \Delta U(T) + \Delta \varepsilon_{ZPE} + \Delta PV - T\Delta S,$$

where ΔG is the reaction Gibbs free energy of each elementary step, $\Delta \varepsilon_{ZPE}$ is the vibrational zero-point energy, T is the temperature (298.15 K), P is the pressure (1 atm), V is the volume of the cell, and ΔS is the change of entropy. ΔZPE was calculated using the vibrational frequency of each step. Lastly, VESTA package was used for structural visualization.[8]

Experimental

Preparation of the α-Ni(OH)₂

Nickel foam was first degreased by immersion in 1 M HCl for 15 minutes to remove surface oxides, followed by ultrasonication in ultrapure deionized water (18.2 MΩ, Millipore) for 5 minutes and in ethanol for 10 minutes. The electrolyte was prepared by dissolving $Ni(NO_3)_2 \cdot 6H_2O$ (Thermo Scientific Chemicals, 99%) in ultrapure deionized water to a concentration of 0.1 M, without any additional additives. The electrodeposition of α-$Ni(OH)_2$ was carried out in a two-electrode configuration using nickel foam as the working electrode and a Pt/Ti plate as the counter electrode. A cathodic current of –50 μA cm^{-2} was applied for 1 hour at room temperature. After deposition, the electrode was rinsed thoroughly with deionized water, dried in ambient air, and subsequently annealed at 100 °C for 1 hour.

Experimental characterization

The crystalline structure of the synthesized catalysts was characterized by X-ray diffraction (XRD) using a Philips X'Pert Panalytical diffractometer equipped with a monochromated Cu $K\alpha_1$ radiation source (λ = 1.5406 Å) operating at 40 kV and 30 mA. Diffraction patterns were collected over a 2θ range of 5–80°. The surface morphology of the catalyst was examined by scanning electron microscopy (SEM) using a JEOL JSM-7800F Prime instrument at the National Institute for Nanomaterials Technology (NINT). High-resolution transmission electron microscopy (HRTEM) and selected area electron diffraction (SAED) were performed using a JEOL JEM-2200FS microscope operated at 200 kV at the NINT. The TEM samples were prepared by dispersing the catalyst in ethanol, followed by drop-casting onto lacey carbon film-coated copper TEM grids. The lattice spacings were measured using DigitalMicrograph software.

The Raman spectra were measured using a Witec(Alpha300R) instrument, with an excitation

wavelength of 532 nm and a 40x objective magnification (Nikon CFI S Plan Fluor ELWD). Each Raman spectrum was obtained as the average of 60 measurements with a collection time of 1s. X-ray photoelectron spectroscopy measurements were conducted using a K-ALPHA+ XPS system (Thermo Fisher Scientific) equipped with Al Kα anode radiation (1486.6 eV) at a setting of 12 kV (72 W) at the Busan Center of KBSI. For the analysis, all spectra's binding energies were calibrated using the reference of C 1s at 284.8 eV.

Electrochemical characterization

Electrochemical tests were conducted in a three-electrode system, using NiOOH as the working electrode, a Pt/Ti plate as the counter electrode, and Hg/HgO (1 M KOH, Dek Research Instrumentation) as the reference electrode, utilizing a Bio-logic potentiostat (VSP 128). All potentials were converted to the reversible hydrogen electrode (RHE) scale ($E_{RHE} = E_{Hg/HgO} + 0.098\ V + 0.059 \times pH$), and were *iR*-corrected to 85% to account for the resistance of the electrolyte. The OER was performed in a 1 M KOH electrolyte, and the UOR was conducted in a 1 M KOH electrolyte with 0.33 M urea added. LSV results were obtained at a scan rate of 5 mV s^{-1}. Tafel slopes were derived from linear relationship between logarithmic current density and potential. Electrochemical impedance spectroscopy (EIS) was conducted at an amplitude of 10 mV, ranging from 100 mHz to 100 kHz. Chronopotentiometry were carried out at a constant current density of 100 mA cm^{-2} over a period of 1 hour. For chronoamperometry, specific potentials of 1.5, 1.6, and 1.7 V RHE. were applied. Ion chromatography (DX-120) was utilized to analyze the by-products formed during the UOR process. Electrochemical impedance spectra were obtained at 1.55 V RHE with an amplitude of 10 mV from 100 kHz to 100 mHz. Operando FT-IR spectra were acquired at each applied potential with a resolution of 4 cm^{-1} using a Nicolet™ iS50 FTIR Spectrometer (Thermo Fisher Scientific).

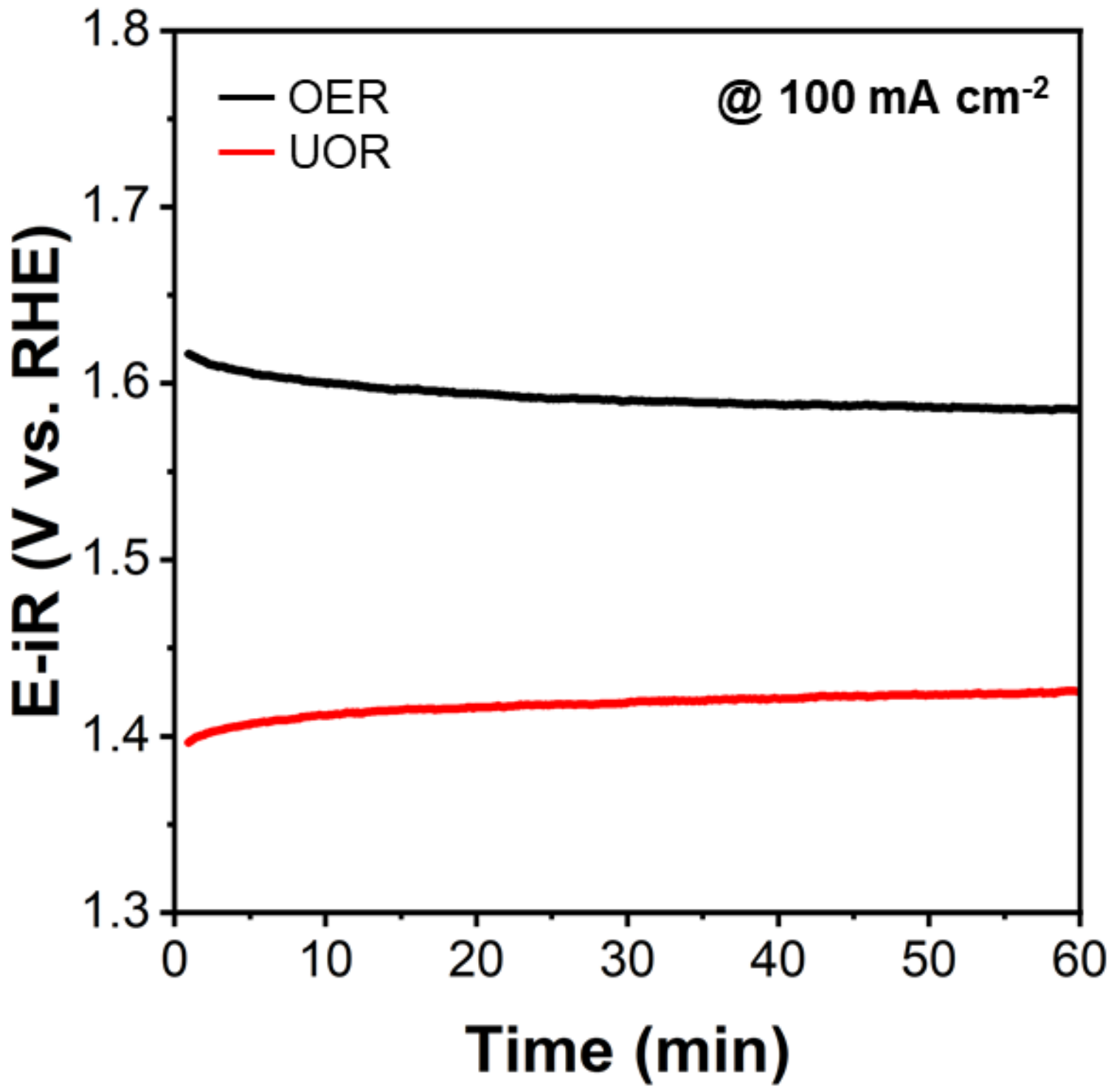

Figure S1. Chronoamperometry measurements for 1 hour at 100 mA cm^{-2} in 1 M KOH and 1 M KOH + 0.33 M urea electrolytes.

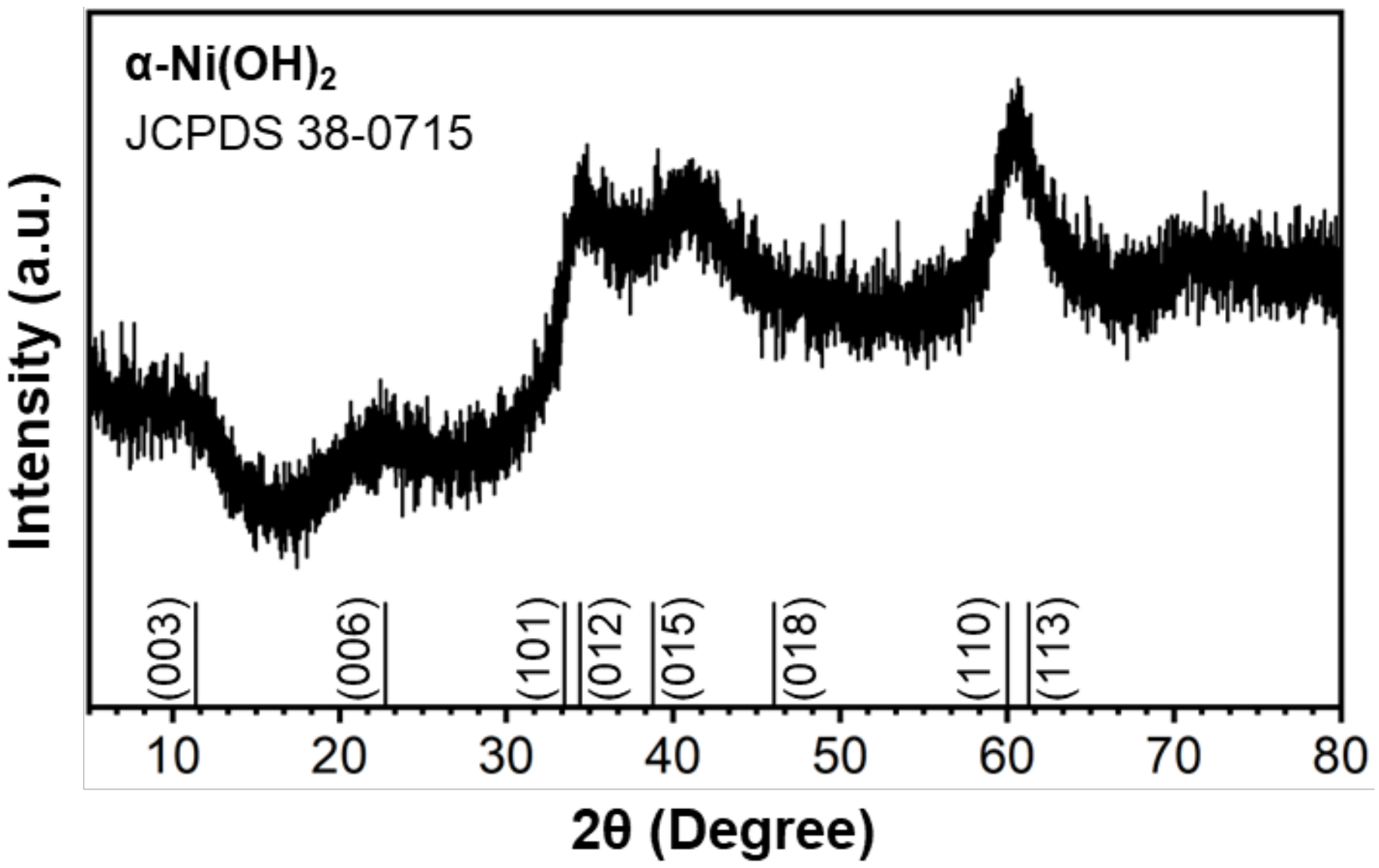

Figure S2. XRD pattern of the electrodeposited nickel hydroxide catalyst, confirming the α-$Ni(OH)_2$ phase.

Table S1. XRD peak positions and assignments for α-$Ni(OH)_2$ (JCPDS: 38-0715).

2theta [deg]	d (Å)	(h k l)
11.35	7.79	(0 0 3)
22.74	3.91	(0 0 6)
33.46	2.68	(1 0 1)
34.41	2.60	(0 1 2)
38.77	2.32	(0 1 5)
45.99	1.97	(0 1 8)
59.98	1.54	(1 1 0)
61.26	1.52	(1 1 3)

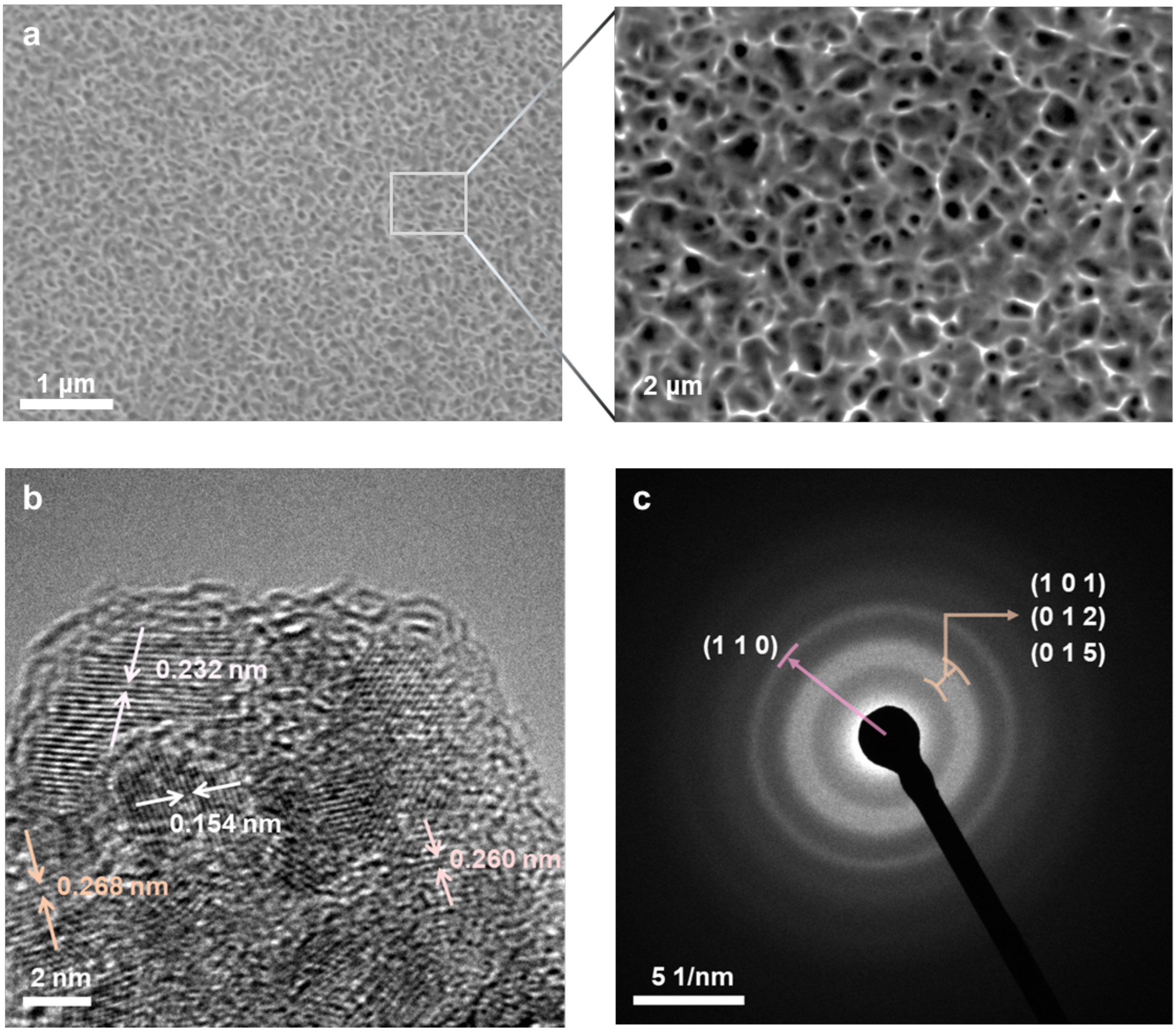

Figure S3. a) SEM images at low and high magnifications, b) high-resolution TEM image, and c) SAED pattern of the electrodeposited nickel hydroxide catalyst.

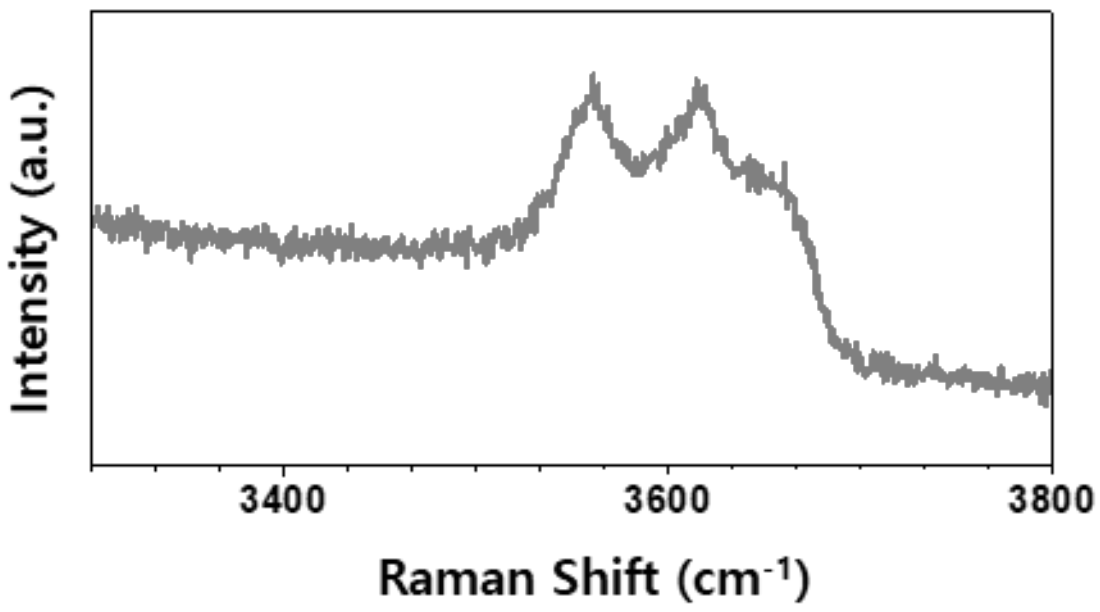

Figure S4. Raman spectroscopy in the O–H stretching region for the electrodeposited nickel hydroxide catalyst.

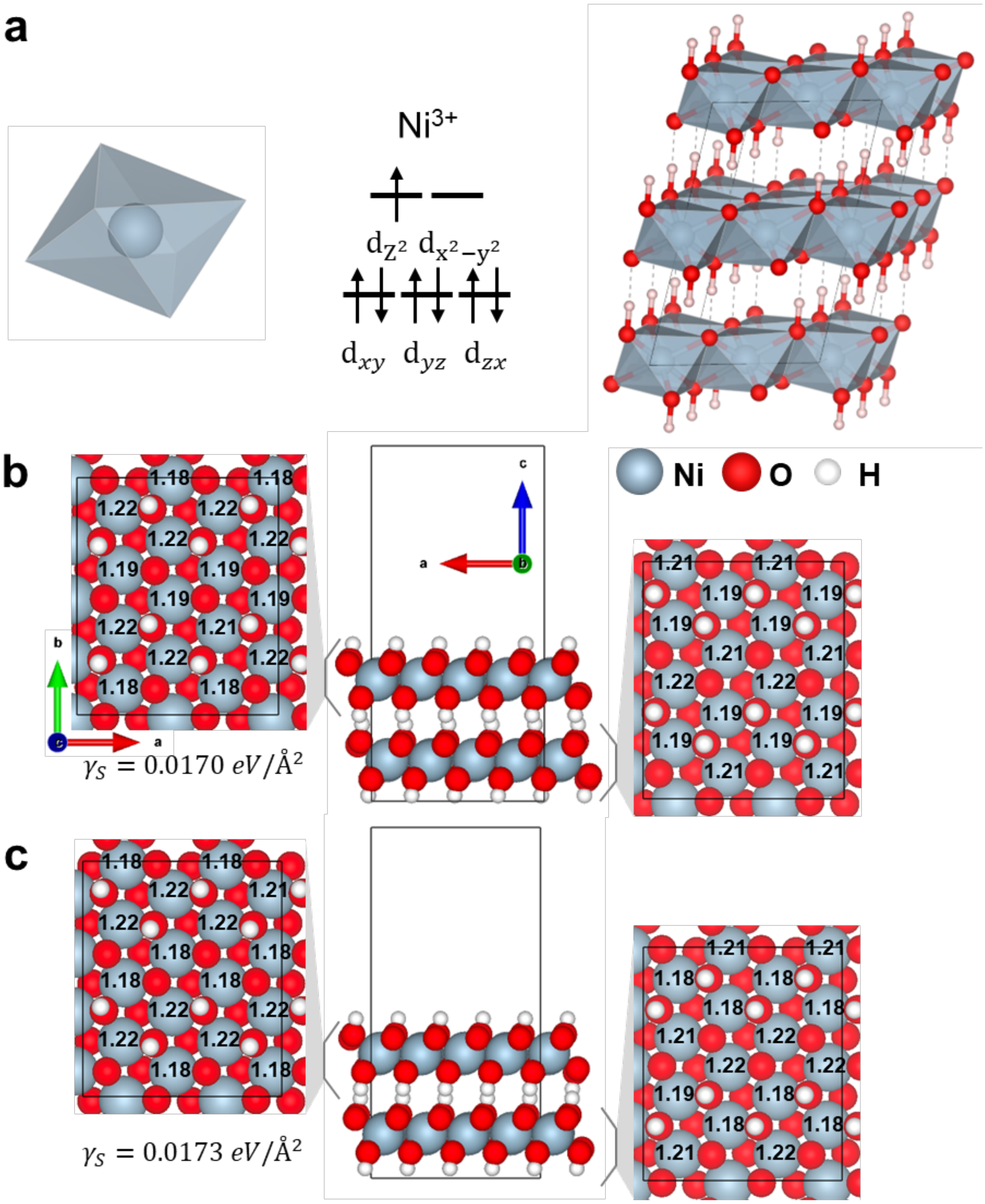

Figure S5. DFT-optimized atomic structures of β-NiOOH (001). a) Pristine bulk model with electronic configuration, b) Termination A with a surface energy of 0.0170 eV/Å^2, and c) Termination B with a surface energy of 0.0173 eV/Å^2. The magnetic moments of all Ni atoms are indicated.

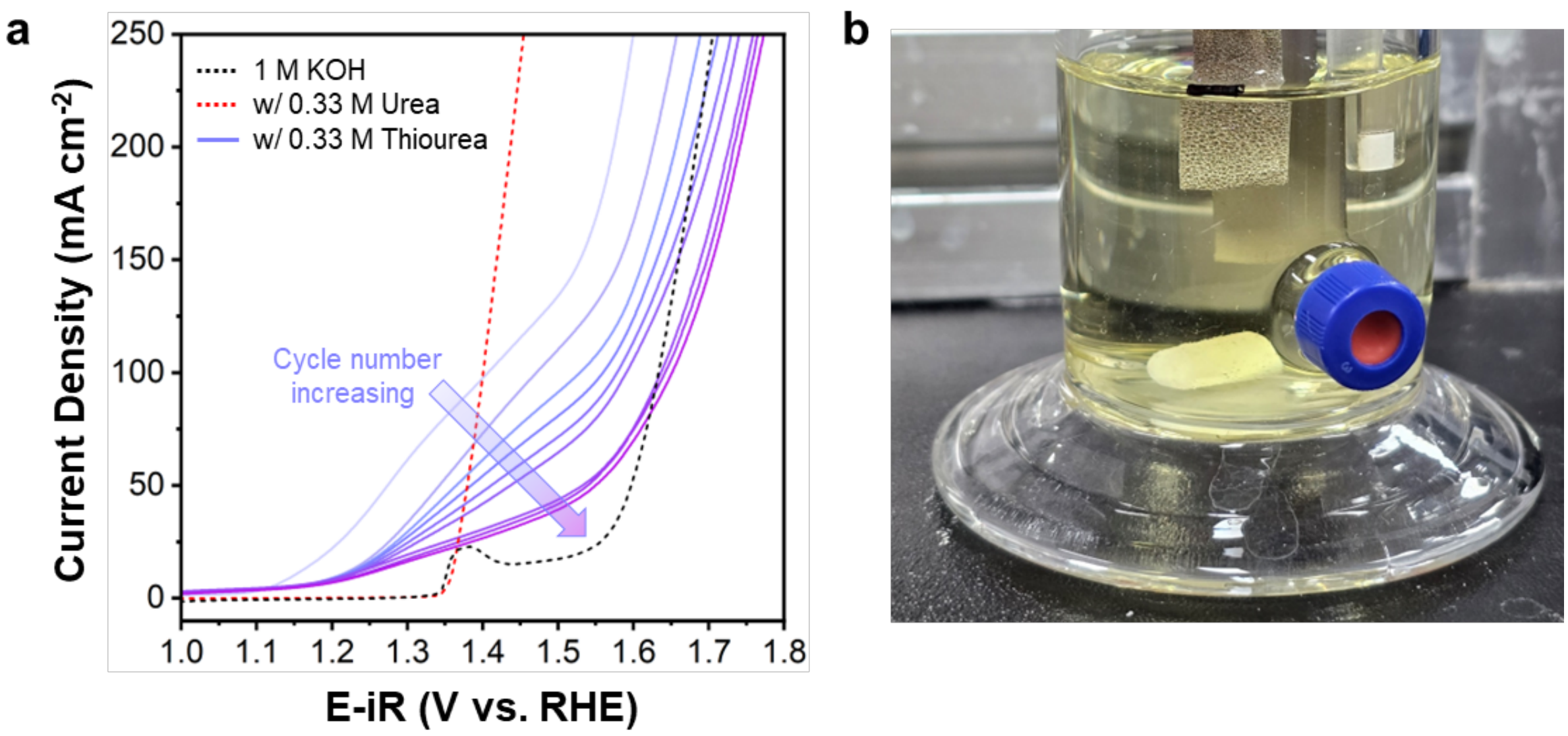

Figure S6. a) Repeated LSV curves in 1 M KOH with 0.33 M thiourea. b) Photograph of the electrolyte after 10 LSVs in the presence of 0.33 M thiourea.

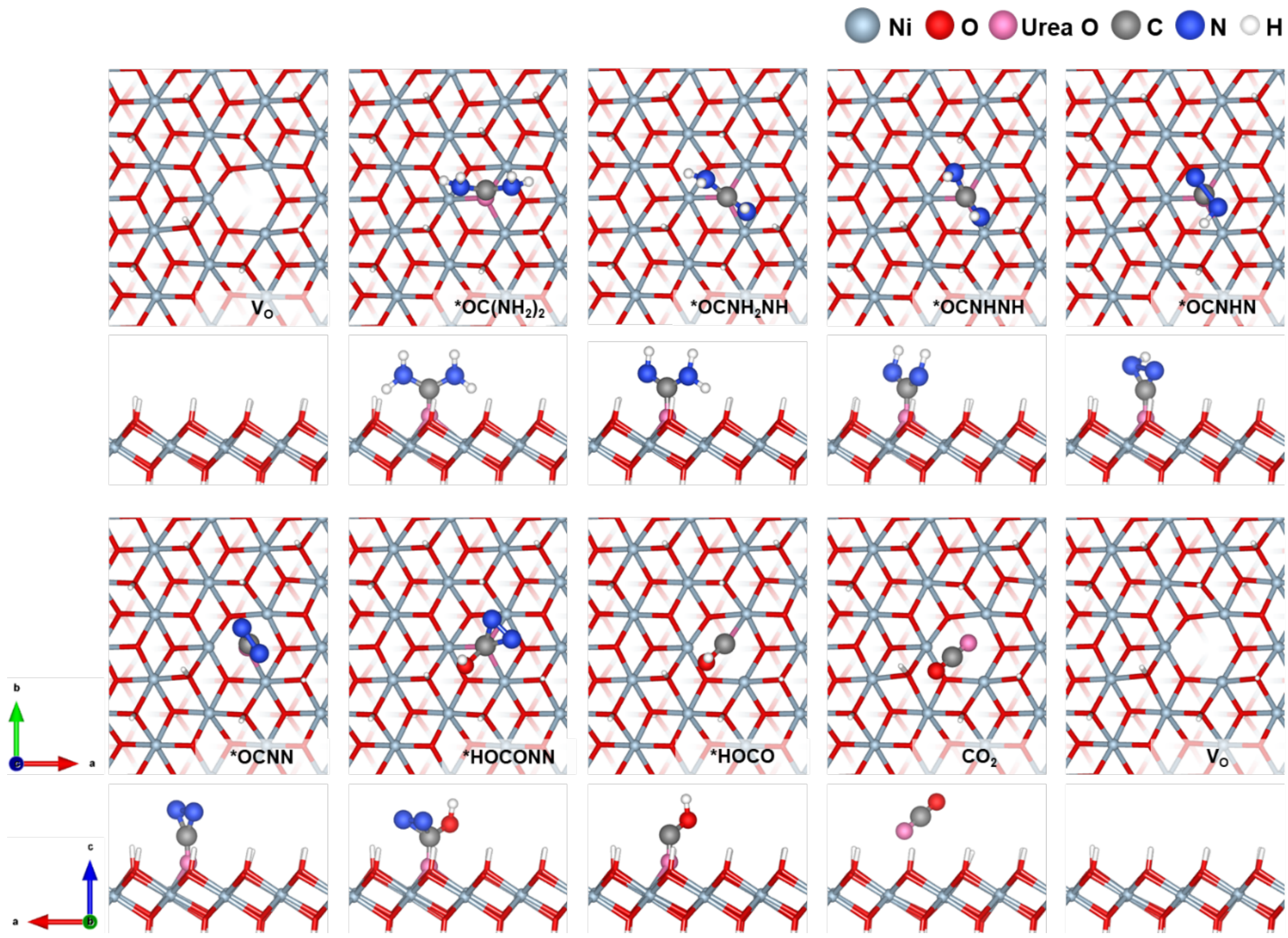

Figure S7. DFT-optimized intermediate configurations in the N_2 and CO_2 formation pathways of urea oxidation: at the oxygen vacancy (V_O) site of β-NiOOH (001)

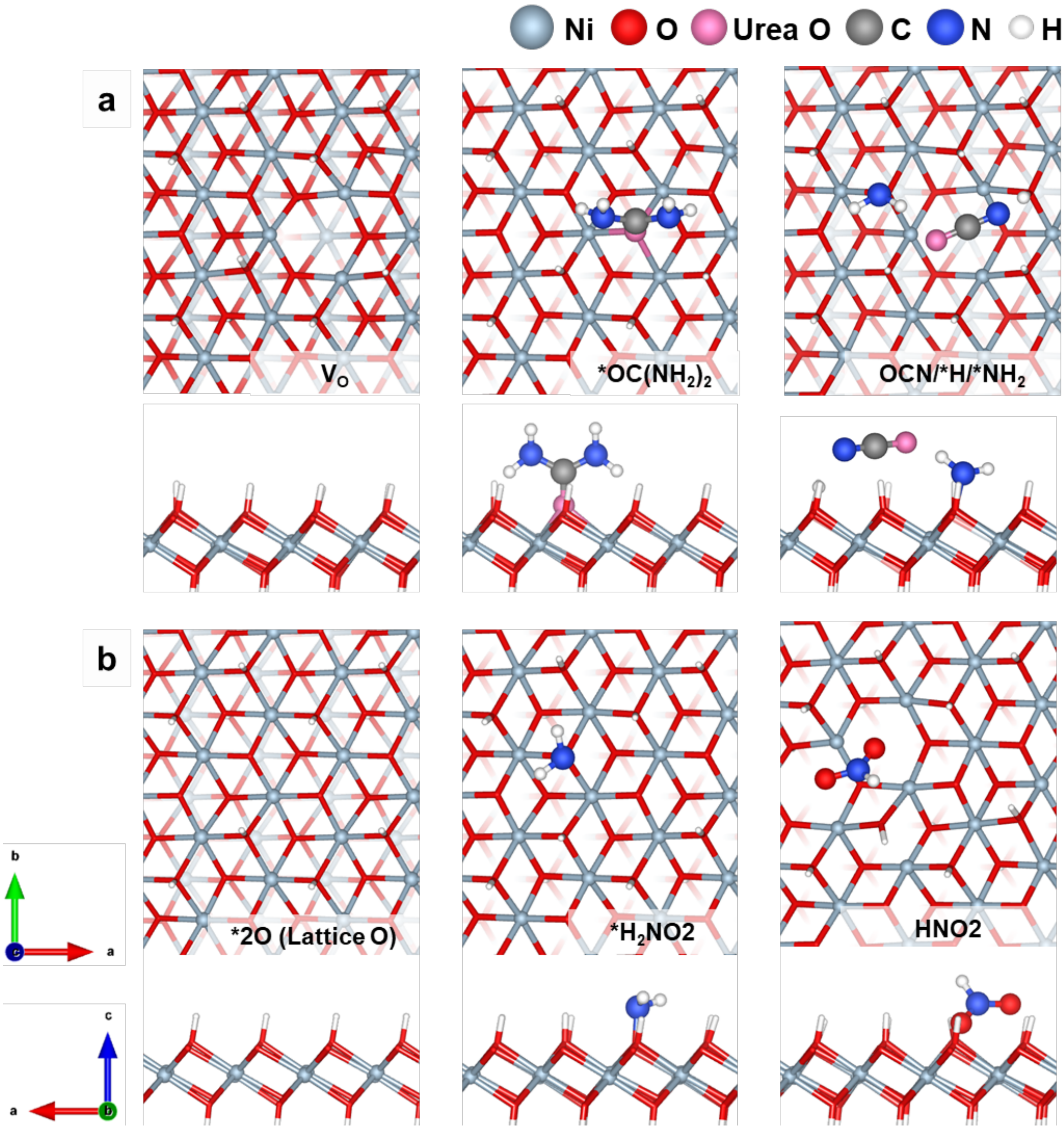

Figure S8. DFT-optimized intermediate configurations in the cyanate and nitrite formation pathways of urea oxidation: a) cyanate formation at the oxygen vacancy (V_O) site b) nitrite formation at the pristine surface, involving lattice oxygen atoms with NH_2 species resulting from the cyanate reaction.

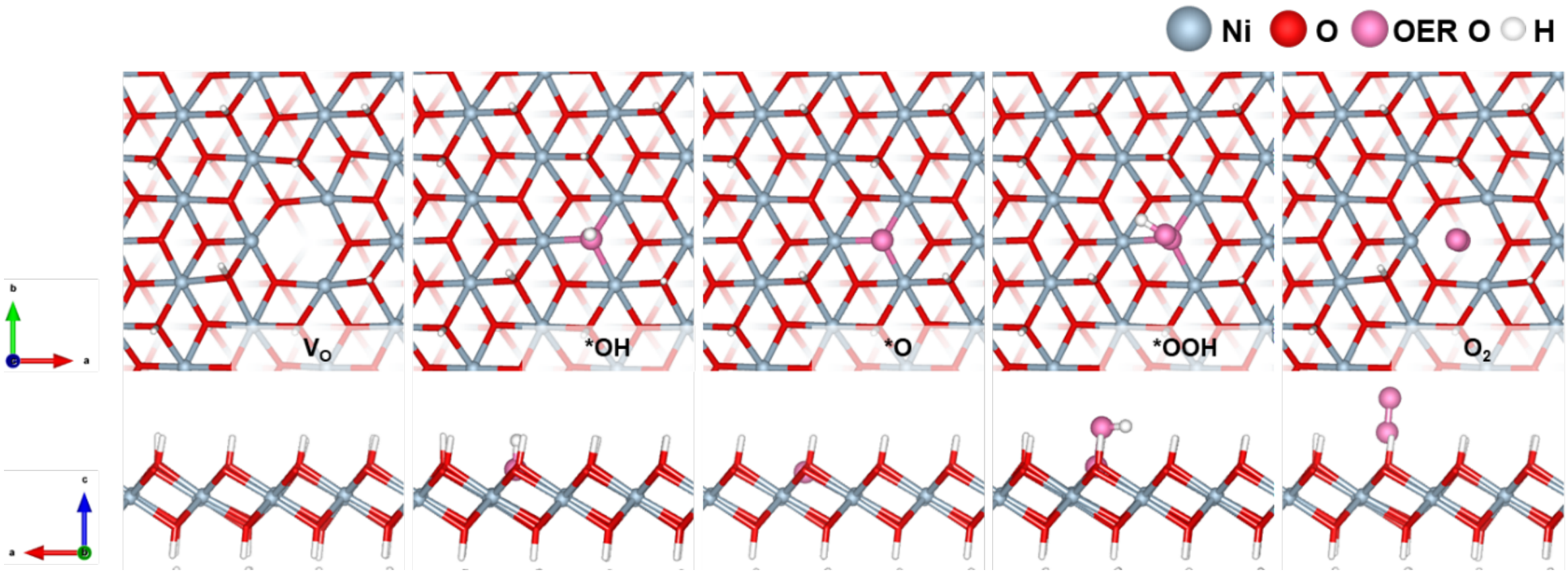

Figure S9. DFT-optimized intermediate configurations in the oxygen evolution reaction (OER) pathway at the oxygen vacancy (V_O) site of β-NiOOH (001).

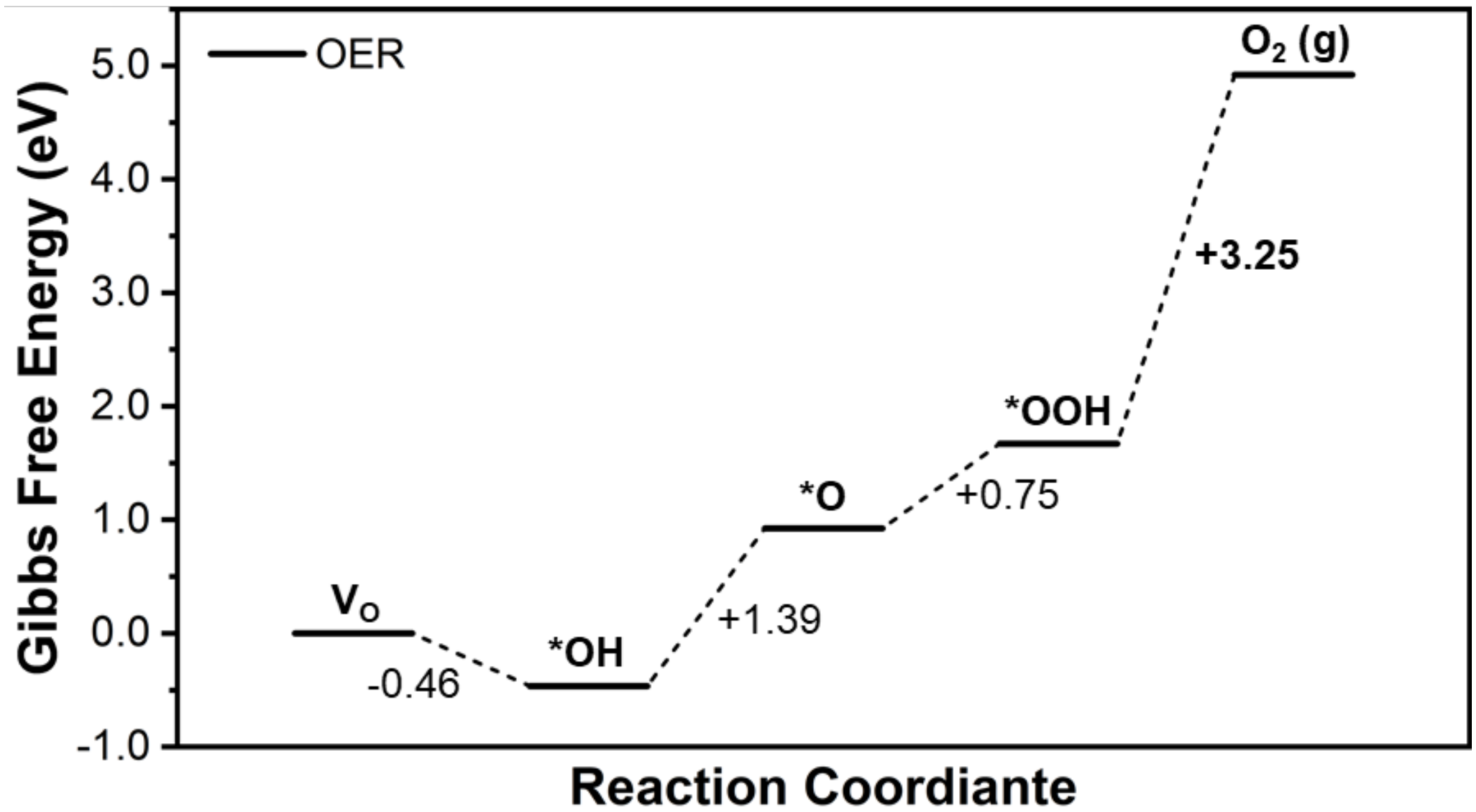

Figure S10. Gibbs free energy diagrams of OER pathway.

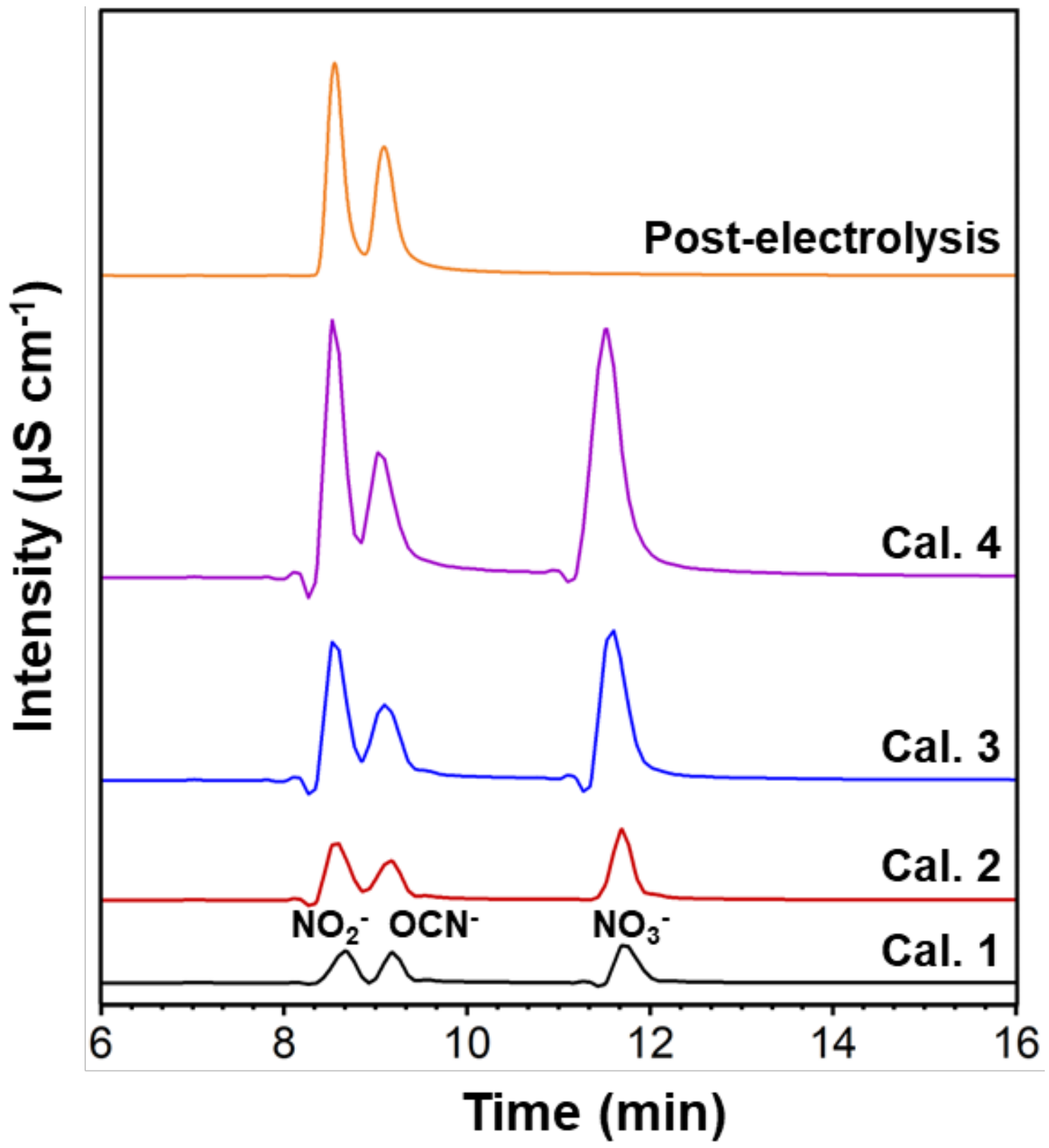

Figure S11. Chromatograms for IC calibration and Post-UOR sample for quantification of NO_2^-, OCN^-, and NO_3^-. Cal. 1-4 corresponds to mixed standards at concentration of 0.2, 0.4, 0.8, and 1.6 mM (for each anion). The post-electrolysis electrolyte collected after 1.6 V for 5 h in 1 M KOH with 0.33 M urea, was analyzed after 100-fold dilution. The chromatogram of post-electrolysis sample shows peaks in coincidence with the NO_2^- and OCN^- standards, whereas NO_3^- was not detected.

Reference

[1] a) G. Kresse, J. Furthmüller, Phys. Rev. B 1996, 54, 11169; b) D. S. Sholl, J.A. Steckel, Density functional theory: a practical introduction, John Wiley & Sons, Hoboken, NJ 2022.

[2] a) J. Wellendorff, K. T. Lundgaard, A. Møgelhøj, V. Petzold, D. D. Landis, J. K. Nørskov, T. Bligaard, K. W. Jacobsen, Phys. Rev. B 2012, 85, 235149; b) S. Grimme, J. Comput. Chem. 2006, 27, 1787.

[3] G. Kresse, J. Hafner, *J. Phys.: Condens. Matter* **1994**, *6*, 8245.

[4] H. J. Monkhorst, J. D. Pack, *Phys. Rev. B* **1976**, *13*, 5188.

[5] W. H. Press, B. P. Flannery, S. A. Teukolsky, W. T. Vetterling, *Numerical Recipes: The Art of Scientific Computing,* Cambridge University Press, New York **1986**.

[6] V. I. Anisimov, J. Zaanen, O. K. Andersen, *Phys. Rev. B* **1991**, *44*, 943.

[7] V. Wang, N. Xu, J.-C. Liu, G. Tang, W.-T. Geng, Comput. Phys. Commun. 2021, 267, 108033.

[8] K. Momma, F. Izumi, J. Appl. Cryst. 2011, 44, 1272.
